# Identification of Evolutionary Trajectories Shared across Human Betacoronaviruses

**DOI:** 10.1093/gbe/evad076

**Published:** 2023-05-23

**Authors:** Marina Escalera-Zamudio, Sergei L Kosakovsky Pond, Natalia Martínez de la Viña, Bernardo Gutiérrez, Rhys P D Inward, Julien Thézé, Lucy van Dorp, Hugo G Castelán-Sánchez, Thomas A Bowden, Oliver G Pybus, Ruben J G Hulswit

**Affiliations:** Department of Biology, University of Oxford, Oxford, United Kingdom; Consorcio Mexicano de Vigilancia Genómica (CoViGen-Mex), Mexico City, Mexico; Department of Biology, Institute for Genomics and Evolutionary Medicine, Temple University, Philadelphia, PA 19122, USA; Department of Biology, University of Oxford, Oxford, United Kingdom; Department of Biology, University of Oxford, Oxford, United Kingdom; Consorcio Mexicano de Vigilancia Genómica (CoViGen-Mex), Mexico City, Mexico; Department of Biology, University of Oxford, Oxford, United Kingdom; Epidemiology of Animal and Zoonotic Diseases, UMR 0346, INRAE, VetAgroSup, Saint-Gènes-Champanelle, France; Department of Genetics, Evolution and Environment, UCL Genetics Institute, University College London, London, United Kingdom; Consorcio Mexicano de Vigilancia Genómica (CoViGen-Mex), Mexico City, Mexico; Programa de Investigadoras e Investigadores por México, Consejo Nacional de Ciencia y Tecnología, Mexico City, México; Division of Structural Biology, Wellcome Centre for Human Genetics, University of Oxford, Oxford, United Kingdom; Department of Biology, University of Oxford, Oxford, United Kingdom; Department of Pathobiology, Royal Veterinary College, London, United Kingdom; Division of Structural Biology, Wellcome Centre for Human Genetics, University of Oxford, Oxford, United Kingdom

**Keywords:** molecular evolution, phylogenomics, convergence, adaptation, betacoronaviruses

## Abstract

Comparing the evolution of distantly related viruses can provide insights into common adaptive processes related to shared ecological niches. Phylogenetic approaches, coupled with other molecular evolution tools, can help identify mutations informative on adaptation, although the structural contextualization of these to functional sites of proteins may help gain insight into their biological properties. Two zoonotic betacoronaviruses capable of sustained human-to-human transmission have caused pandemics in recent times (SARS-CoV-1 and SARS-CoV-2), although a third virus (MERS-CoV) is responsible for sporadic outbreaks linked to animal infections. Moreover, two other betacoronaviruses have circulated endemically in humans for decades (HKU1 and OC43). To search for evidence of adaptive convergence between established and emerging betacoronaviruses capable of sustained human-to-human transmission (HKU1, OC43, SARS-CoV-1, and SARS-CoV-2), we developed a methodological pipeline to classify shared nonsynonymous mutations as putatively denoting homoplasy (repeated mutations that do not share direct common ancestry) or stepwise evolution (sequential mutations leading towards a novel genotype). In parallel, we look for evidence of positive selection and draw upon protein structure data to identify potential biological implications. We find 30 candidate mutations, from which 4 (codon sites 18121 [nsp14/residue 28], 21623 [spike/21], 21635 [spike/25], and 23948 [spike/796]; SARS-CoV-2 genome numbering) further display evolution under positive selection and proximity to functional protein regions. Our findings shed light on potential mechanisms underlying betacoronavirus adaptation to the human host and pinpoint common mutational pathways that may occur during establishment of human endemicity.

**Significance**

Identifying adaptive convergence is intimately linked to the possibility of predicting evolutionary trajectories in viruses relevant to global health. In this light, we undertook a comparative approach to find evidence of adaptive convergence across betacoronaviruses capable of a sustained human-to-human transmission (HKU1, OC43, SARS-CoV-1, and SARS-CoV-2). Our methodology involved the development of a pipeline used for identifying mutations putatively denoting homoplasy and or stepwise evolution that are also evolving under positive selection, and with potential biological implications drawn from protein structural data. Coupled with future experimental data and ongoing genomic surveillance, our results raise the possibility of predicting how the evolutionary trajectory for SARS-CoV-2 may develop as the virus establishes itself as endemic to humans.

## Introduction

Understanding the mutational processes that lead to adaptation in RNA viruses is crucial for developing effective control strategies. Due to their high mutation rates and small genomes, RNA viruses often display rapid evolution. However, the vast majority of mutations are either purged through purifying selection or are selectively neutral (Loewe and Hill 2010). Only a small proportion of these may contribute to adaptive evolution and be consequently fixed through positive selection (Ohta 1973; Kosakovsky Pond et al. 2012). For most viral genomes, the mutational pathways leading to adaptation are further constrained by functional and evolutionary limitations, such as epistasis, which refers to the adaptive dependence of a given mutation on the genetic background in which it appears (Dolan et al. 2018). Therefore, viral evolutionary trajectories are often limited and may exhibit recurrent mutational patterns indicative of adaptive convergence, especially when applied to independent virus populations that share ecological niches (Gutierrez, Escalera-Zamudio, and Pybus 2019).

The OC43 and HKU1 embecoviruses and the SARS-CoV and SARS-CoV-2 sarbecoviruses are four betacoronavirus species capable of sustained human-to-human transmission. OC43 and HKU1 were introduced into the human population through independent zoonotic events estimated to have occurred at least 50 years ago and are associated with mild respiratory disease (Su et al. 2016). In contrast, SARS-CoV and SARS-CoV-2 were independently introduced more recently, causing severe pandemic outbreaks (Li et al. 2005b; Vijaykrishna et al. 2007; Andersen et al. 2020; Boni et al. 2020; Banerjee et al. 2021). In 2002, SARS-CoV spread to more than 20 countries, causing a short-lived outbreak characterized by sustained human-to-human transmission (Cheng et al. 2007). Although its circulation was eventually halted, the virus displayed evidence of adaptation to the human population (Chinese SARS Molecular Epidemiology Consortium 2004). Almost two decades later, SARS-CoV-2 spread globally, resulting in the current pandemic, despite a low rate of adaptive change recorded during the early stages of the outbreak (van Dorp et al. 2020; MacLean et al. 2021). The continuous circulation of OC43 and HKU1 within the human population at a global scale has been accompanied by ongoing host-specific adaptation. This is now also evident for SARS-CoV-2, exemplified by the constant emergence of novel virus lineages across time and space, with sublineages now reflecting regional endemic patterns (O’Toole et al. 2021).

As SARS-CoV-2 becomes established in humans, it will continue to adapt to overcome the selective pressures exerted by the collective immune response of the human population (Kissler et al. 2020). We hypothesize that adaptive convergence may occur across distantly related betacoronaviruses circulating within the same ecological niche, specifically the human host. To test this, we undertook a comparative analysis to search for evidence of shared mutational pathways between established human-endemic embecoviruses and emerging sarbecoviruses, with a focus on emerging mutations observed in SARS-CoV-2. We developed a methodological pipeline that allows for the identification of nonsynonymous mutations (rendering amino acid substitutions) likely associated with adaptive convergence across multiple virus species. Firstly, we detected amino acid substitutions shared across virus taxa, displaying putative evidence of homoplasy or stepwise evolution. Secondly, we assessed whether these substitutions were positively selected and contextualized their location to functional regions of viral proteins. Following our pipeline, we initially detected 30 candidate amino acid substitutions displaying evolutionary patterns denoting putative homoplasy and/or stepwise evolution. We subsequently identified four of these (sites 18121 [nsp14/27], 21623 [spike/21], 21635 [spike/25], and 23948 [spike/796], in SARS-CoV-2 genome coordinates) as positively selected, and proximal to functional surfaces in nsp14 (Ma et al. 2015) and the spike (S) protein. Our results provide a molecular-level context for common evolutionary trajectories that betacoronaviruses may undergo during their adaptation to the human host.

## Results

### Patterns of Genetic Variability Observed in Human-Infecting Betacoronaviruses

**Fig. 1.**
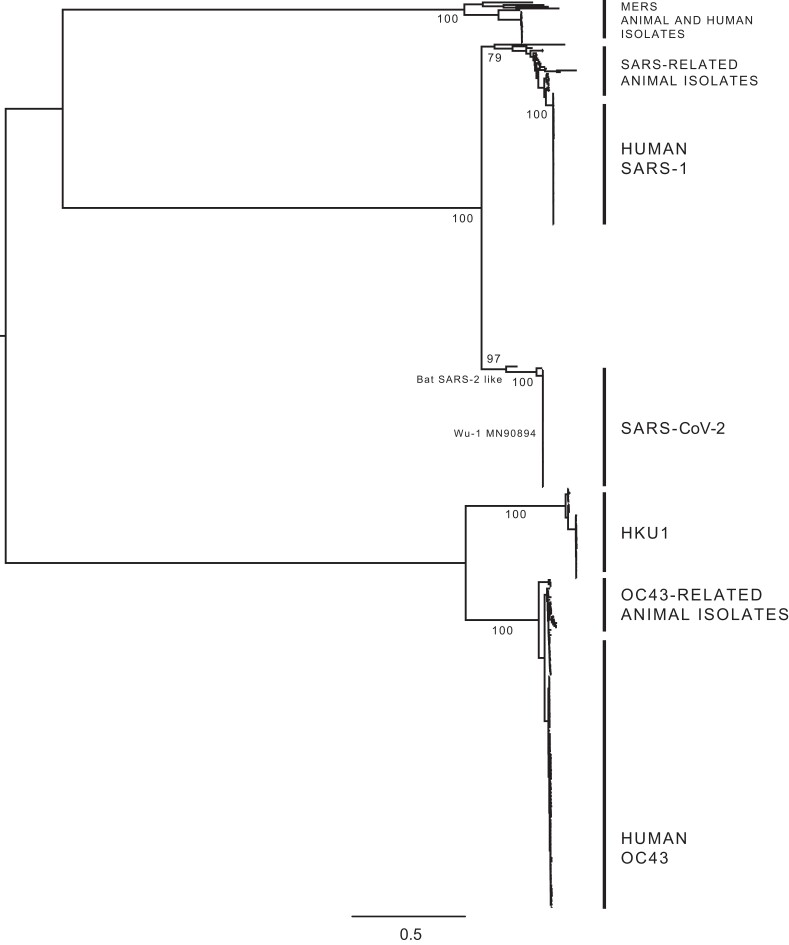
Phylogenetic tree of human-infecting betacoronaviruses. The expanded tree estimated from the Orf1ab + S alignment comprising 1,455 sequences (see Methods section 6, Supplementary Material online), summarizing the phylogenetic pattern observed for four distantly related human-infecting betacoronaviruses: HKU1, OC43, SARS-CoV-1, and SARS-CoV-2. MERS and related virus sequences were included in the tree for rooting purposes only. Both the *Embecovirus* subgenus (HKU1 and OC43 and related viruses) and the *Sarbecovirus* subgenus (SARS-CoV-1 and SARS-CoV-2 and related viruses) are indicated, showing the positioning of the most closely related virus genome sequences derived from animal isolates (when available). The different genotypes identified for the HKU1 (A, B, and C) and for the OC43 (A–H) are shown in supplementary Data 1, Supplementary Material online.

We performed phylogenetic analyses of human-infecting betacoronaviruses using an alignment of the Orf1ab and S viral genes (see Methods sections 1 and 2, Supplementary Material online). The tree shown in figure 1 provides a comprehensive picture of the evolutionary relationships among the four betacoronavirus species studied here, consistent with previously published phylogenies of the genus (Woo et al. 2006; Woo et al. 2010; Oong et al. 2017; Zhu et al. 2018; Bedford 2021). Our analysis confirms four well-supported clades formed by virus sequences belonging to the *Embecovirus* (HKU1, OC43, and related viruses) and *Sarbecovirus* (SARS-CoV, SARS-CoV-2, and related viruses) subgenera (ICTV et al. 2017). To further validate divergence patterns at a deeper node level, we compared individual clades (subtrees within our trees) with species-specific phylogenies. We were also able to verify the divergence patterns described for the distinct HKU1 (A-C) and OC43 (A-H) genotypes (Woo et al. 2006; Oong et al. 2017) (supplementary Data 1, Supplementary Material online). Therefore, our phylogenetic reconstructions validate the evolutionary relationships among these four distantly related betacoronaviruses.

**Fig. 2.**
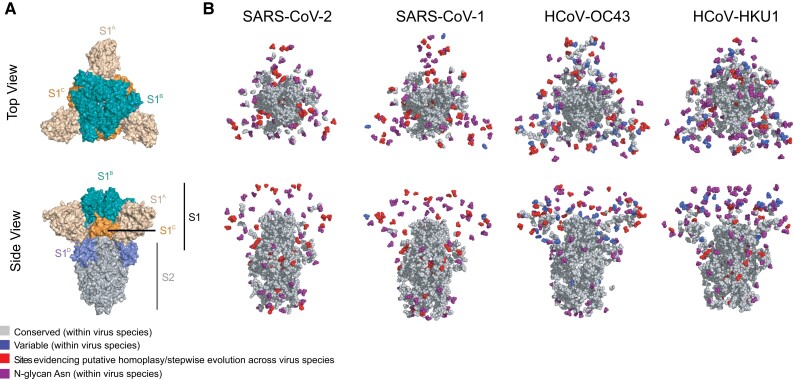
Distribution of conserved/variable sites with S across different virus species (A) Top-down (upper panel) and side view (bottom panel) of a cartoon representation of the multidomain architecture of the trimeric SARS-CoV-2 S ectodomain (PDB: 6VXX). The cartoon displays the S1 subunit dispalying different protein domains (S1A, S1B, S1C and S1D) and the S2 subunit. (B) Top-down and side views of spherebased representations of trimeric S protein ectodomains for the viruses studied here: SARS-CoV-2 (PDB: 6VXX), SARS-CoV-1 (PDB: 6ACC), OC43 (PDB: 6OHW) and HKU1 (PDB: 5I08). The sphere-based representation shows conserved (residues present ≥99% of all sequences) and variable sites (residues present in ≥1% of all sequences) across virus species. Variable sites identified as denoting homoplasy or stepwise evolutionary patterns are shown separately (see Methods section 3). The asparagine residues of N-linked glycosylation sequons are indicated in purple.

We then analyzed the proportion of codon sites (from the total number of polymorphic sites identified), corresponding to nonsynonymous mutations shared between different embeco- and sarbecovirus species (i.e., those present in any of the sarbecovirus clades and also in HKU1 and/or OC43). Derived from the Orf1ab + S alignment (comprising a total of 8,962 sites), we identify approximately 2% (205 sites) as shared. Within the Orf1a region (4,774 sites), 2.7% of these (129 sites) were identified as shared. Within the Orf1b region (2,623 sites), only 0.9% (25 sites) were further identified as shared. The Orf S region (1,457 sites) displayed the highest proportion of shared mutations (3.2%, 48 sites). When analyzing genetic variation patterns within single virus species, we observed a high degree of sequence conservation (>91% identity) across the Orf S of all virus species. Conserved sites were predominantly located in the membrane proximal S2 domain, although variable sites were mostly found within the membrane distal S1 subunit (fig. 2). The predominance of variable sites within S1 compared with S2 was most evident for embecoviruses, and less so for sarbecoviruses, suggesting for a differential adaptation stage relative to the human host environment, evidenced by a lower degree of genetic divergence observed in Orf S in the sarbecoviruses.

We further analyzed the genetic variation across virus species, focusing on the Orf S region. As previously noted for other coronaviruses (Hulswit et al. 2016), we found that Orf S exhibited a higher proportion of variable sites relative to conserved (for definitions, see Methods section 3, Supplementary Material online). Specifically, only 16% of homologous sites within the Orf S alignment were conserved, although the remaining 84% were variable (supplementary Data 2, Supplementary Material online). The S2 subunit of Orf S contained the highest proportion of conserved sites, presumably due to shared functional constraints of the viral membrane fusion machinery across coronavirus species (Li 2016). Conversely, the S1 subunit displayed a higher number of variable sites, particularly within the S1^A^ domain (also known as the N-terminal domain or [NTD]). We found that the S1^B^ domain did not display any conserved sites across virus species, likely due to differences in receptor engagement between embeco- and sarbecoviruses. Specifically, embecoviruses use the S1^A^ domain to interact with sialoglycan-based receptors, although sarbecoviruses use their S1^B^ domain to bind to angiotensin-converting enzyme 2 (ACE2) (Hulswit et al. 2019; Lan et al. 2020). Finally, we identified that the conserved R residue at site 685 corresponding to the S1/S2 cleavage site (numbering according to the SARS-CoV-2 protein, codon sites 23615–23617) is shared across and within virus species (supplementary Data 2, Supplementary Material online), reflecting a conserved proteolytic maturation mechanism of the spike protein (Millet and Whittaker 2015).

### Sites Displaying Evidence of Homoplasy and/or Stepwise Evolution

**Fig. 3.**
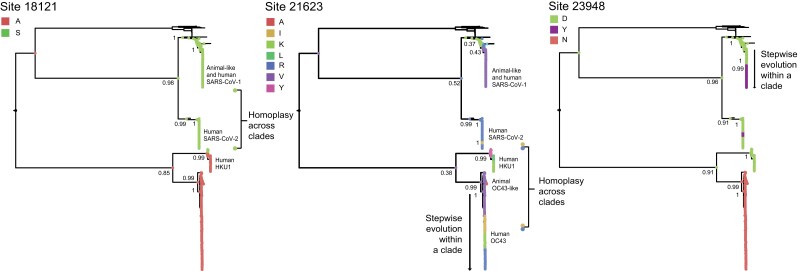
Reconstruction of amino acid evolution at selected sites. Maximum clade credibility (MCC) trees showing the evolutionary trajectories for different amino acid states observed for three illustrative sites (18121, 21623 and 23948) that (i) display evidence of homoplasy and/or stepwise evolution, (ii) show evidence of positive selection, and (iii) are proximal to regions of established protein function. The reconstructions of ancestral states for these sites show different amino acid states at nodes (represented by circles). The posterior probability for a given amino acid state occurring at a given node of interest is indicated. Sites 18121 display evidence of homoplasy across virus lineages, site 21623 shows evidence of both homoplasy across species and stepwise evolution within single virus species (i.e. OC43), and site 23948 shows evidence of stepwise evolution within single virus species (i.e. SARS-CoV-1), and also of homoplasy across virus species (i.e. SARS-1 and SARS-CoV-2).

Although not all nonsynonymous mutations putatively displaying homoplasy and/or stepwise evolution may arise from positive section, such mutational patterns are most likely to result from adaption (Escalera-Zamudio et al. 2020; Stern et al. 2017; Gutierrez et al. 2019). Thus, among the nonsynonymous mutations identified as shared across virus species, we further searched for those displaying putative evidence for homoplasy and/or stepwise evolution (supplementary Text 1, Supplementary Material online) using our pipeline (Methods section 3, Supplementary Material online). After visual validation, we confirmed that 30 sites (representing 0.3% within the Orf1ab + S alignment) display evolutionary patterns indicative of homoplasy and/or stepwise evolution (see supplementary Text 2 and figs. S2 and S3, Supplementary Material online). Two of these were found within Orf1a, nine within Orf1b, and 19 within Orf S (table 1). The evolutionary trajectories for different amino acid states observed for three illustrative sites (18121, 21623, and 23948, further displaying evidence of evolution under positive selection and of being proximal to regions of established protein function [see the following results sections]) are highlighted below (fig. 3). The amino acid evolution patterns observed for all other sites are available in supplementary Data 3, Supplementary Material online.

**Table 1:** Potentially Relevant Sites across Human-Infecting Betacoronaviruses

			Mutations observed (global tree)						Confirmed in resampled trees					
SARS-CoV-2 genome coordinates^a^	ORF	Protein/residue^a^	Ancestral LinA	OC43	HKU1	Ancestral LinB	SARS-CoV-1	SARS-CoV-2^c^	Expanded	1400 SARS-CoV-2	Homoplasy (H)/stepwise evolution (SWE)	Selection across species (method, *P*-value)^a^,^b^	Selection in SARS-CoV-2 (recent amino acid changes)^d^	Epitopes^e^
2557	Orf1a	nsp2 585	P	P	S	S	A/T	P/S	Yes	Conserved in SARS-CoV-2	H/SWE			0
7478	Orf1a	nsp3 1587	N	S/N	N	N	T	N	Yes	Conserved in SARS-CoV-2	H		PSS, N → S/D (OC43-like and new state)	0
16189	Orf1b	nsp12 917	D	D	E/D	E	E	E	Yes	Conserved in SARS-CoV-2	H/SWE	Overall negative selection (FEL 0.02)		1
17809	Orf1b	nsp13 525	V	V	V/I	I	I	I	Yes	Conserved in SARS-CoV-2	H			0
**18121**	**Orf1b**	**nsp14 28**	**A**	**A**	**A/S**	**S**	**S**	**S**	Yes	Conserved in SARS-CoV-2	**H**	**Different overall positive selection (CF 0.022)**		**1**
18334	Orf1b	nsp14 100	D	D	E/D	E	D	E	Yes	Conserved in SARS-CoV-2	H/SWE	Overall negative selection (FEL 0.004)		0
18442	Orf1b	nsp14 136	K	K	K/R	R	R	R	Yes	Conserved in SARS-CoV-2	H			0
19048	Orf1b	nsp14 338	A	G/A	G	A	A	A	Yes	Conserved in SARS-CoV-2	H/SWE	OC43 branch (MEME 0.035)		0
20344	Orf1b	nsp15 243	Q	Q	H/Y	H	H	H	Yes	Conserved in SARS-CoV-2	H/SWE			2
20554	Orf1b	nsp15 313	N	N	S/N	S	S	S	No	Conserved in SARS-CoV-2	H	Overall negative selection (FEL 0.04)		0
21400	Orf1b	nsp16 249	A	A	T/S	S	S	S	Yes	Conserved in SARS-CoV-2	H/SWE			2
21614	Orf S	S1 18	F	F/I/L	I	L	F	L	Yes	Variable in SARS-CoV-2	H/SWE		PSS, L → F (OC43 and SARS-CoV-1-like)	1
**21623**	**Orf S**	**S1 21**	**V**	**R/V/K/I**	**K/Y/L**	**R**	**V**	**R/I**	Yes	Conserved in SARS-CoV-2	**H/SWE**	**HKU1, OC43 and SARS-2 branches (MEME 0.047)**	**NSS, R** **→** **I/K/T (OC43 and HKU1-like and new state)**	**1**
**21635**	**Orf S**	**S1 25**	**V**	**P/V/S/L/H**	**V/I**	**P**	**N**	**P/S**	Yes	Conserved in SARS-CoV-2	**H/SWE**	**HKU1, OC43 and SARS-2 branches (MEME 0.048)**	**NSS, P** **→** **S and L (OC43-like)**	0
21800	Orf S	S1 81	K	K	Q/K	D	G/D	D	Yes	Variable in SARS-CoV-2	SWE		PSS, D → Y/A/G (SARS-CoV-1-like and new states)	0
21863	Orf S	S1 102	Y	F/I/T	Y	I	V	I	Yes	Conserved in SARS-CoV-2	H/SWE		PSS, I → V (SARS-CoV-1-like)	0
21920	Orf S	S1 120	V	V	V/I	V	I	V	Yes	Conserved in SARS-CoV-2	H/SWE			0
21926	Orf S	S1 122	T	T	N/T	N	N	N	Yes	Conserved in SARS-CoV-2	H/SWE	Overall negative selection (FEL 0.002)	NSS	0
22004	Orf S	S1 149	N	N/K	K/I	N	G	N	Yes	Conserved in SARS-CoV-2	H		NSS, N → D (new state)	0
22124	Orf S	S1 189	D	T/D/N	D	H	H	N	Yes	Conserved in SARS-CoV-2	H/SWE	OC43 branch (MEME 0.008)	NSS	0
22553	Orf S	S1 332	N	D/N	D/N	N	N	N	Yes	Conserved in SARS-CoV-2	H/SWE			1
23048	Orf S	S1 497	S	A/G/S	D/S	G	G	G	Yes	Variable in SARS-CoV-2	H/SWE	HKU1 branch (MEME 0.044)		2
**23948**	**Orf S**	**S2 796**	**D**	**N**	**D**	**D**	**Y/D**	**Y/D**	**D/N discrepancy**	Variable in SARS-CoV-2	**SWE**	**Different overall positive selection (CF 0.031)**	**PSS, D** **→** **Y/G/H (SARS-CoV-1-like and new states)**	**0**
24614	Orf S	S2 1018	V	V	V/I	I	I	I	Yes	Conserved in SARS-CoV-2	H		NSS	1
24620	Orf S	S2 1020	F	F	F/A/L	A	A	A	Yes	Conserved in SARS-CoV-2	H		PSS, A → S/V (new states)	2
24632	Orf S	S2 1024	Q	Q	L/R	L	L	L	Yes	Conserved in SARS-CoV-2	H/SWE			2
24863	Orf S	S2 1101	T	T	H/S	H	S	H	Yes	Conserved in SARS-CoV-2	H/SWE		NSS, H → Y (new state)	1
25037	Orf S	S2 1159	Q	Q	Q/H	H	H	H	Yes	Conserved in SARS-CoV-2	H		NSS, H → Y (new state)	0
25166	Orf S	S2 1202	D	D/Y	D/E	E	E	E	Yes	Conserved in SARS-CoV-2	H		PSS, E → Q/G (new states)	0
25247	Orf S	S2 1230	V	V	V/M	M	M	M	Yes	Conserved in SARS-CoV-2	H		PSS, M → I/T/L (new states)	1

a Positions indicate the start of the codon for reference genome Wuhan-Hu-1 (NC_045512.2). Sites in bold refer to those highlighted in the results section.

b Sites/branches scored under MEME/FEL and Contrast-FEL (CF); CF tests for differences are selective pressures between clades.

c Representing virus diversity sampled as of May 2021.

d Representing viral diversity sampled as of December 2022 available from https://observablehq.com/@spond/sars_cov_2_sites.

e Potential T cell epitopes derived from HLA class I and HLA-DR SARS-CoV-2 binding peptides (Campbell et al. 2020; Nelde et al. 2021).

Derived from the global, expanded and the resampled SARS-CoV-2 trees (Methods section 1, 3, and 6 and Text 3, Supplementary Material online), our results show that site 18121 (codon 18121–18123 in Orf1b, corresponding to amino acid state “S” in nsp14 in SARS-CoV-2 numbering) is homoplasic between HKU1 genotype B and the sarbecoviruses (table 1, fig. 3, and supplementary Data 3, Supplementary Material online). Comparably, site 21623 (codon 21623–21626 in Orf S, corresponding to amino acid state “R” in S) was identified as homoplasic between SARS-CoV-2 and OC43 genotypes D, F, G, and H. This site also displayed evidence for stepwise evolution within a single virus clade (OC43), exemplified by the sequential amino acid replacement pattern of V → I → K → R (fig. 3).

For site 23948 (codon 23948–23950 in Orf S, corresponding to residue 796 in S), initial observations based on the global tree revealed that amino acid state “D” was present in all virus species, except for OC43 (displaying amino acid state “N”). However, when replicating our analyses (expanded tree), the distribution of amino acid state “D” was now found present in some embecoviruses (including OC43 but excluding HKU1) and most sarbecoviruses. These discrepancies are likely due to alignment uncertainty across genome regions of highly divergent virus taxa. Nonetheless, based on consensus protein sequences and structural comparison, the structural contextualization of amino acid 796 and adjacent sites confirmed the presence of “D” in SARS-CoV-1, SARS-CoV-2, and HKU1, and “N” in OC43, (supplementary fig. S6, Supplementary Material online). Thus, amino acid state “D” at site 23948 shows evidence of homoplasy between the SARS-CoV-1, SARS-CoV-2, and HKU1.

For this same site (23948), an additional amino acid change from “D” to “Y” was identified as homoplasic between some SARS-CoV-1 and SARS-CoV-2 sequences (data derived from the global, expanded and the resampled SARS-CoV-2 trees) (table 1). For SARS-CoV-2, amino acid state “Y” emerged and was lost repeatedly during the early stage of the pandemic (represented by independent minor clusters that quickly became extinct). However, following emergence and global spread of the B.1.1.529 virus lineage (Omicron variant of concern [VOC] and descending sublineages), amino acid state “Y” replaced amino acid state “D,” displaying a predominant trend associated with the dominance of the B.1.617.2 lineage (Delta VOC and descending sublineages) (table 1, fig. 3, and supplementary Data 3, Supplementary Material online) (also confirmed by independently sampled SARS-CoV-2 data available up to December 2022: https://nextstrain.org/groups/neherlab/ncov/global?c=gt-S_796).

### Quantifying the Effects of Positive Selection

The dN/dS estimates we obtained across complete virus genomes and upon specific coding regions (see Methods section 4, Supplementary Material online) indicate that positive selection is acting upon the Orf1ab and Orf S of SARS-CoV-2, compared with other viruses studied here. Specifically, the effect of episodic diversifying selection was detected upon 5/14 nonrecombinant fragments (three in Orf1b and two in Orf S; for details, see https://observablehq.com/@spond/beta-cov-analysis). Using the Contrast-FEL method to detect the effect of a differential selection across branches separating lineages (see Methods section 4, Supplementary Material online), we found 36 sites (0.4%) evolving under differential selective pressure across distinct virus clades. Furthermore, we found 0.7% of all sites (67 codons within the Orf1ab + S alignment) to be evolving under episodic diversifying positive selection (scored under MEME with a *P* ≤ 0.05 as positively selected sites [PSS]) (supplementary table S1, Supplementary Material online). In contrast, we found 5% of all sites (461 codons within the Orf1ab + S alignment) to be evolving under pervasive negative selection (scored under FEL with a *P* ≤ 0.05 as negatively selected sites [NSS]). We subsequently mapped the identified PSS and NSSs onto the SARS-CoV-2 S protein structure (Methods section 5, Supplementary Material online). We observe that out of a total of 22 PSSs, 18 locate within the S1 subunit (11 in S1^A^, 5 in S1^B^, 1 in S1^C^, and 1 in S1^D^ domains), although the remaining four mapped onto the S2 subunit. Conversely, out of a total of 82 of NSSs, 46 locate within S1 (18 in S1^A^, 21 in S1^B^, 3 in S1^C^, and 4 in S1^D^), although the remaining 36 mapped onto S2 (supplementary fig. S7, Supplementary Material online).

From the 30 nonsynonymous mutations we identify as displaying evolutionary patterns putatively denoting homoplasy and/or stepwise evolution (table 1), sites 19048, 21623, 21635, 22124, and 23048 were further scored as PSS (under different methods). Sites 21623 and 21635 were inferred as PSSs along ancestral branches leading to the HKU1, OC43, and SARS-CoV-2 clades. Sites 19048 and 22124 were inferred as PSSs along the OC43 ancestral branch, although 23048 was inferred as a PSS along the HKU1 ancestral branch (table 1 and supplementary table S1, Supplementary Material online). Further analysis under the branch and site model in the MEME method (Methods section 4, Supplementary Material online) revealed site 18121 to be evolving under positive selection for the HKU1 clade/branch (relative to the sarbecoviruses), in agreement with our observations made on putative homoplasy detected for this site between HKU1 genotype B, SARS-CoV-1, and SARS-CoV-2 (table 1 and fig. 3). Similarly, site 23948 was also inferred to be evolving under positive selective for the SARS-CoV-1 branch, relative to other virus clades (supplementary table S1, Supplementary Material online).

For validation, we compared our results with selection analysis available for independently sampled SARS-CoV-2 genome data available as of December 2022 (https://observablehq.com/@spond/evolutionary-annotation-of-sars-cov-2-covid-19-genomes-enab) (Kosakovsky Pond). Of the 30 mutations we identify, 16 of these are currently scored as PSS or NSSs, with 13 of these mapping directly onto potential T cell epitopes derived from HLA class I and HLA-DR binding peptides in SARS-CoV-2 (Nelde et al. 2021; Campbell et al. 2020) (table 1). Additionality, up to December 2022, sites 7478, 21614, 23948, 24620, and 25166 were detected as evolving under positive selection, although sites 21635, 24863, and 25037 were detected as evolving under negative selection.

### Contextualization of Mutations Using Protein Structural and Functional Information

We then mapped the 30 mutations identified onto the corresponding protein structures. Below, we focus on four exemplary sites (18121, 21623, 21635, and 23948) that meet the three criteria of displaying evidence of homoplasy and/or stepwise evolution, showing evidence of evolution under positive selection, and being proximal to regions of established protein function. A description for the other 26 identified mutations is available in the supplementary Text 4 and table S2, Supplementary Material online.

### Site 18121 in Orf1ab

**Fig. 4.**
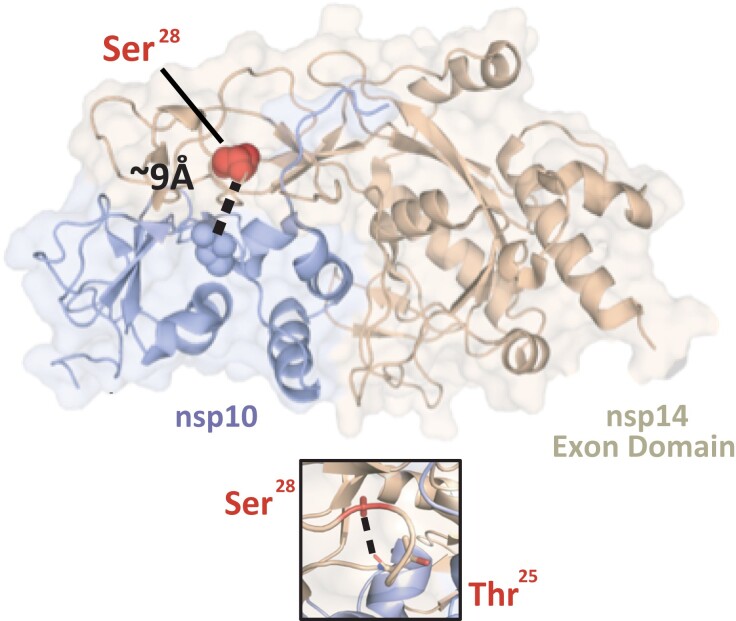
Residue Ser_28_ of nsp14 is situated near the nsp14-nsp10 interface. Cartoon representation of the SARS-CoV-1 nsp14-nsp10 protein complex (PDB: 5C8S) with Ser_28_ (corresponding to site 18121 in SARS-CoV-2 genome coordinates) shown as a red sphere. This residue is located within the nsp14 ExoN domain (cream) and is approximately 9 Å from the interface with nsp10 (the proximal nsp10 residue Cys41 was used to calculate the distance and is indicated as a sphere). The distance between nsp14's Ser_28_ and nsp10's Cys41 is annotated and indicated by a dashed black line. Zoomed-in panel: detailed representation of the intra-nsp14 hydrogen-bond between the side chain of Ser_28_ and the main chain of Thr_25_ (identified with the PISAebi server). The side chain of Ser_25_ andThr_25_ are indicated in ball and sticks according to their corresponding atoms (C, O, N,). The hydrogen-bond is indicated as a dashed line.

Site 18121 is located within the Orf1ab gene and corresponds to an “S” to “A” mutation at residue 28 within the exonuclease domain of the nsp14 protein (numbering according to the SARS-CoV protein) (fig. 4 and supplementary table S2, Supplementary Material online). Nsp14 is involved in the 5′-capping of viral mRNA and is essential for viral mRNA transcription (Ma et al. 2015). The “S” to “A” mutation within this region is expected to result in the loss of an intraprotein hydrogen bond and potentially modulates the protein–protein interaction (fig. 4) (assessed under PISAebi; http://www.ebi.ac.uk/pdbe/prot_int/pistart.html) (Krissinel and Henrick 2007).

### Sites 21623 and 21635 in S1

**Fig. 5.**
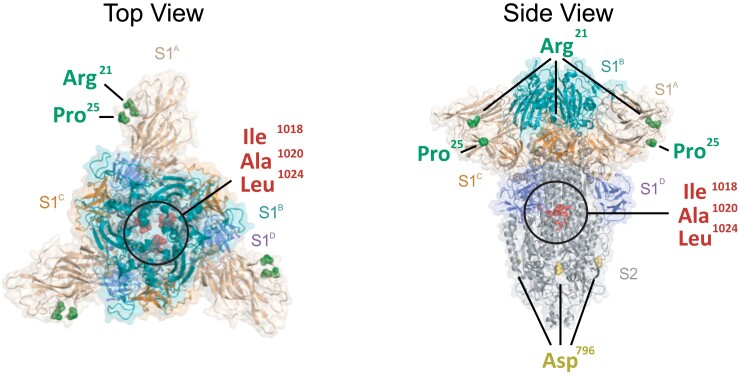
Mapping of mutations exhibiting homoplasy onto the S protein structure of SARS-CoV-2. Top-down (left) and side view (right) of a cartoon representation of the multidomain architecture of the trimeric SARS-CoV-2 S ectodomain (PDB: 6VXX). The S2 subunit is shown whilst the S1 ectodomain is highlighted to show the S1_A_, S1_B_, S1_C_, and S1_D_ domains, following figure 3. Homoplasic mutations that co-localize to known functional surfaces (see Supplementary Table 2) are indicated in the structure: Arg_21_ (corresponding to site 21623 in SARS-CoV-2 genome coordinates), Pro_25_ (site 21635), Asp_796_ (site 23948), Ile_1018_ (site 24614), Ala_1020_ (site 24620) and Leu_1024_ (site 24632). All representations are shown with a transparent protein surface for clarity purposes.

The S1 subunit mediates attachment of the virus to the host cell (Li 2016). Human-infecting embecoviruses bind to glycan-based cell receptors via two hydrophobic pockets within the S1^A^ region of the protein (Hulswit et al. 2019; Tortorici et al. 2019), although the receptor-binding site for human-infecting sarbecoviruses is located within the S1^B^ domain of the protein (Li et al. 2005a, 2005b; Lan et al. 2020; Shang et al. 2020). Both SARS-CoV and SARS-CoV-2 recognize the ACE2 molecule to enter the host cell, despite limited conservation among contact residues within the RBD of these virus species (Li et al. 2005a, 2005b; Lan et al. 2020). Site 21623 displays several nonsynonymous mutations (“R,” “V,” “K,” and “I”) mapping to residue 29 within the S1^A^ domain of the S1 subunit. Site 21635 also shows multiple nonsynonymous mutations (“P,” “V,” “S,” “L,” and “H”) mapping to residue 33 in S1^A^. For the OC43 S protein, this corresponds to a loop neighboring the hydrophobic pockets in S1^A^ instrumental for receptor recognition (fig. 5), and changes within this region may potentially modulate receptor affinity (Hulswit et al. 2019). The mutational patterns observed at these sites putatively denote homoplasy/stepwise evolution and evidence of positive selection (table 1) and are therefore congruent with antigenic drift shaping the evolution of human-endemic coronaviruses (Kistler and Bedford 2021). In SARS-CoV-2, mutations in both these sites (residue 29 and 33) have been observed for two VOCs (B.1.351 and P.1, “Beta,” and “Gamma”) (Faria et al. 2021; Tegally et al. 2021). Even though sarbecoviruses engage the ACE2 receptor via domain S1^B^, these residues locate to the “NTD supersite,” serving as epitope for multiple of neutralizing antibodies (Kemp et al. 2021).

### Site 23948 in S2

The S2 subunit of the betacoronavirus S protein contains the fusion machinery, responsible for merging the viral envelope with the host cell membrane to facilitate delivery of the viral genome into the target cell. This process is driven by the fusion peptide, which anchors the virus to the host membrane, and requires cleavage of the S protein by host cell proteases at the S1-S2 junction (consensus RRAR|S in SARS-CoV-2) and at the S2’ cleavage site (R|S, located immediately upstream of the fusion peptide in the S2 subunit) (Li 2016, Millet and Whittaker 2015). Site 23948 displays a nonconservative amino acid replacement “D” to “Y” (identified as homoplasic between some SARS-CoV and SARS-CoV-2 sequences) at residue 796 of the S2 subunit, located immediately upstream of the S2’ cleavage site (table 1 and supplementary table S2, Supplementary Material online). This residue locates within a loop crucial for the release of the fusion peptide, exhibiting some variability across betacoronavirus species (supplementary fig. S6, Supplementary Material online). Our observations suggest that the apparent relaxed local constraints at this site may facilitate cleavage activation by securing loop accessibility. Perhaps consistently, the corresponding protein region in the HKU1 structure remains unresolved (Kirchdoerfer et al. 2016).

## Discussion

In this study, we searched for signatures of adaptive convergence across distantly related human-infecting betacoronaviruses, represented by shared nonsynonymous mutations that putatively denote homoplasy and/or stepwise evolution, further ranked according to their selective relevance, and to their proximity to protein regions of known function. The majority of the mutations we observe locate to the receptor binding region of the S protein (i.e., S1 subunit), although a smaller proportion of these were found within nonstructural proteins encoded by Orf1ab (site 18121 in the exonuclease domain of nsp14 and site 20344 in the endonuclease domain of nsp15). Our in silico analyses revealed four genomic sites (18121, 21623, 21635, and 23948) that display cumulative evidence of 1) a mutational pattern putatively denoting homoplasy and/or stepwise evolution, 2) evolution under positive selection, and 3) being structurally proximal to regions of known protein function. Below, we discuss our findings in light of three key evolutionary processes: antigenic drift, epistasis, and adaptive convergence.

The host humoral immune response is an evolutionary force driving viral antigenic drift. In the case of betacoronaviruses, this is reflected by cumulative mutations in the S protein (particularly within the S1 subunit) that may allow frequent reinfections of the host population (Kistler and Bedford 2021; Yewdell 2021; Forni et al. 2021). In agreement with this observation, the emergence of some SARS-CoV-2 lineages (particularly VOC) has been associated with high levels of infection in pre-exposed human populations across different geographic regions (as an example on P.1, see Faria et al. 2021). Our results evidence antigenic drift upon the S1 subunit of distinct betacoronaviruses as a major component of the adaptation process to the human host environment, further evidenced by Orf S also being the least conserved genome region across distinct virus species (Li 2016). On the other hand, mutations found within Orf1ab could have a potential impact on viral fitness related to an enhanced replication efficacy in the human host (Menachery et al. 2017). As the evolution of Orf1ab is also driven by immune responses such as cytokine signaling cascades and antigen presentation (Wang et al. 2015; Taefehshokr et al. 2020; Hackbart et al. 2020; Yuen et al. 2020), these mutations may also be the result of concerted selective pressure(s), following that single mutational changes can have pleiotropic effects on distinct viral phenotypes and fitness components (de Wilde et al. 2018).

Identifying adaptive convergence raises the possibility of predicting mutational pathways in viruses important to global health (Gutierrez et al. 2019). When applied to SARS-CoV-2, our results reveal that some of the mutations we had initially identified as potentially relevant back in May 2021 (see Escalera-Zamudio et al. 2021) had already been observed in other betacoronaviruses that circulate endemically in humans (table 1), and some now display dominant trends in SARS-CoV-2 (as analyzed up to December 2022). For example, amino acid state “R” at residue 21 of the S protein (sites 21623) (https://nextstrain.org/groups/neherlab/ncov/global?c=gt-S_21) and “P” at residue 25 (site 21635) (https://nextstrain.org/groups/neherlab/ncov/global?c=gt-S_25) have dominated across time. Moreover, mutation “D” to “Y” observed at residue 796 of the S protein (site 23948) has proven to be a successful mutational pathway, evidenced by the replacement of amino acid state “D” (previously observed for the B.1.617.2 lineage, Delta VOC, and descending sublineages) by “Y” (now observed for the B.1.1.529 lineage, Omicron VOC, and descending sublineages) (https://nextstrain.org/groups/neherlab/ncov/global?c=gt-S_796). Of interest, mutations at residue 796 of the S protein have been linked to the emergence viral variants that display reduced susceptibility to neutralizing antibodies (Kemp et al. 2021).

Epistasis is thought to have played a central role in the emergence of human-infecting betacoronaviruses (Holmes and Rambaut 2004). However, inferring epistasis across diverging viruses is difficult given the functional differences between homologous genes and proteins. Through our methodological approach, we cannot measure epistasis per se, but we can aim to identify adaptive convergence and subsequently discuss its possible effects. Thus, our results indirectly provide support for epistasis, in the sense that if the same amino acid changes are observed in different virus species, then associated epistatic interactions are expected to be shared. This is of particular importance when considering the potential role of epistasis in antigenic drift, where the combined effect of independent mutations could contribute to antigenic escape (Rochman et al 2022). In the context of our findings, sites 21623 and 21635 are presumed to be involved in the antigenic drift of embecoviruses. As these residues are in close proximity to each other (displaying a linked evolution), these could thus reflect epistatic interactions. Nevertheless, within the SARS-CoV-2 S1^B^-ACE2 interface, epistasis seems to play a limited role, as the effect of multiple mutations seems to be additive rather than epistatic (Rochman et al. 2022; Zahradník et al. 2021; Starr et al. 2022).

The mutational spectrum of SARS-CoV-2 is known to be impacted by the human host apolipoprotein B mRNA editing enzyme (APOBEC) family (Di Giorgio et al. 2020). The activity of APOBEC induces C → U/T mutations in the viral genome through a cytidine deaminase activity, likely resulting in a high degree of apparent homoplasy reflected in emerging mutations across distinct virus subpopulations (De Maio et al. 2020; Worobey et al. 2020; Wang et al. 2020). Relative to more commonly used strategies for identifying homoplasy within single virus species, our methodology poses an alternative approach that aims to identify homoplasy across and within virus taxa, represented by shared mutations most likely fixed under an evolutionary scenario driven by selection (see supplementary Text 5, Supplementary Material online). Given that candidate mutations are observed over longer evolutionary times, this approach represents a useful tool to decrease the likelihood of erroneously scoring mutations as homoplasic (such as those resulting from mutational biases inherent to the SARS-CoV-2 genome evolution).

However, identifying adaptive convergence faces several important limitations. First, the methodology we use is conservative, as it is based on strict homology. In this context, we only consider sites robustly identifiable as homologous that can be traced back to ancestral nodes with confidence (consequently excluding highly divergent genes). Therefore, our approach may result in an underestimation of sites that may putatively denote adaptive convergence across highly divergent viruses. Moreover, a limited virus genome sampling across time and space (in particular for HKU1 and SARS-CoV-1), coupled with a relatively low genetic diversity observed for SARS-CoV-2 (Rausch et al. 2020), further restricts the potential to identify shared mutations across virus species (van Dorp et al. 2020). In addition, there is some uncertainty associated with the mutations identified, as (though unlikely given cumulative evidence derived from different methodological approaches) it is not possible to rule out that some of these may still derive from biological processes other than adaptation (such as founder effects, mutational hitchhiking, linkage, and toggling at hypervariable sites) (Kosakovsky Pond et al. 2012; Delport et al. 2008; De Maio et al. 2021; Wang et al. 2020; Simmonds 2020). Finally, although our analysis provides insights into coronavirus evolution in humans, our approach renders us unable to identify mutations that may result from host switching events. This is due to analyses on nodes representing ancient host switching events (Corman et al. 2018) being constrained by long divergence times, differences in mutation rates across virus taxa in different animal hosts, mutational saturation, and a considerable undersampling of betacoronaviruses circulating in nonhuman hosts (Holmes and Rambaut 2004; De Maio et al. 2021).

In this sense, additional/future experimental data could help reveal the impact of mutations on viral fitness. However, performing such studies may be difficult, as these concern potential gain-of-function experiments. Alternatively, enhanced genomic surveillance of betacoronaviruses infecting the human population and of those circulating in other animal host may confirm whether the mutational pathways we identify here represent evolutionary trajectories on which betacoronaviruses converge in their adaptation process to the human host.

## Material and Methods

### Initial Data Collation

When this manuscript was first deposited as a preprint (May 2021) (Escalera-Zamudio et al. 2021), complete genomes for all HKU1, OC43, and SARS-CoV-1 viruses sampled across different geographical regions and time were downloaded from the Virus Pathogen Resource (ViPR-NCBI 2021) (supplementary Data 4, Supplementary Material online). Sequences were removed if meeting any of the following criteria: 1) being >1,000 nt shorter than the full genome length, 2) being identical to any other sequence, or 3) if showing >10% of site were ambiguities (including N or X). A total of 53 HKU1, 136 OC43, and 40 SARS-CoV-1 sequences were initially retained for analyses. For SARS-CoV-2, to better reflect an early zoonotic process into the human population (MacLean et al. 2021), we originally aimed to limit the genetic diversity of the sampled virus population to the first wave of infection recorded during the pandemic. For this, ∼23,000 full genomes sampled worldwide before May 2021 available in the GISAID platform (GISAID 2021) were downloaded and aligned as part of an initial public data set provided by the COG-UK consortium (COG-UK 2021) (supplementary Data 4, Supplementary Material online). To make local analyses computationally feasible, the original SARS-CoV-2 data set was randomly subsampled to ∼5% of its original size, keeping the earliest genomes, and further reducing the data set under the quality criteria stated above. In total, 1,120 SARS-CoV-2 sequences were retained. For all virus species considered, we focused only on genomes derived from human cases, in order to reflect host-specific adaptation processes.

### Phylogenetic Analyses

Only the main viral ORFs (Orf1ab and S) were used for further phylogenetic analyses, as these are homologous among the four viral species studied, and encode proteins essential to certain stages of the virus life cycle (i.e., replication and entry). For each virus species, individual ORFs (codons) were extracted and aligned as translated amino acid sequences using MAFFT v7.471 (to be then reverted to codons again) (Katoh and Standley 2013). Individual alignments were concatenated to further generate species-specific concatenated Orf1ab + S alignments. The concatenated alignments were then combined to generate a global alignment comprising all virus species that was realigned again at an amino acid level using a profile-to-profile approach following taxonomic relatedness (Wang and Dunbrack 2004). The final alignment was reverted to codon sequences as input for all further analyses. The global alignment comprised in total 1,314 sequences and 26,883 sites.

Maximum likelihood phylogenies were estimated for the individual and global codon alignments using RAxML v8 (Stamatakis 2015), under a general time reversible nucleotide substitution model and a gamma-distributed among-site rate variation (GTR + G). Branch support was assessed using 100 bootstrap replicates. All trees were midpoint rooted, although general phylogenetic patterns observed among these distantly related virus species were validated by comparing to previously published phylogenies (Woo et al. 2010; Zhu et al. 2018; Lau et al. 2011; Oong et al. 2017; Woo et al. 2006; Bedford 2021). Recombination is known to be common among betacoronaviruses (Oong et al. 2017; Woo et al. 2006; Su et al. 2016), including SARS-CoV-2 (Gutierrez et al. 2022; Turakhia et al. 2022). However, recombinant sequences were not removed at this step, as it was important to detect potentially recombinant isolates that could display relevant mutations. Putative recombinant sequences were eventually removed for subsequent analyses (when identified, see Methods sections 6 and 7, Supplementary Material online).

### Identifying Homoplasy and/or Stepwise Evolution

Following the pipeline described by Escalera and Golden (Escalera-Zamudio et al. 2020), variable sites across different virus taxa were identified within the global alignment as those displaying nonsynonymous mutations (rendering amino acid changes) occurring in at least ≥1% of the sampled sequences. Variable sites were extracted by masking columns across the alignment showing identical sites and at least 50% gaps, followed by the “Find Variations/SNPs” function used to compare each site with consensus sequences generated under a 95% threshold with Geneious Prime v2020.0.4 (Kearse et al. 2012). A total of 6,681 variable sites were identified and used to infer ancestral amino acid state reconstructions onto the nodes/internal branches of the global tree (see Methods section 2, Supplementary Material online, above). This was done using TreeTime (Sagulenko et al. 2018) under a maximum likelihood (ML) approach (RAS-ML) using a time-reversible model (GTR) for state transitions. The genetic variability observed within leaves/tips of the tree was deliberately excluded, in order to only analyze changes occurring within nodes or internal branches. In parallel, conserved sites were identified as those present in ≥99% of the sampled virus sequences. Conserved sites were extracted by reversing the “variable site masking,” to obtain only identical sites identified across the global alignment (supplementary Data 2, Supplementary Material online).

The resulting 6,681 “Ancestral Reconstruction Trees” (named here ARTs) were then classified under a computational algorithm developed to sort mutational patterns based on whether or not they support homoplasy and/or stepwise evolution. Briefly, homoplasy can occur within nodes of single clade or across clades, in which the same amino acid change must be present in at least one internal node of any given clade, and in another internal node of the same/another clade. Clades with the same amino acid states must not share direct common ancestry. Conversely, stepwise evolution is represented as sequential mutations occurring at the same sites within a single clade. Any given site scored under putative “stepwise evolution” must display changes between at least two different states (A → B), but without any immediate reversion (B → A). A description of the definitions used here for homoplasy and/or stepwise evolution is available as supplementary Text 1 and figure S1, Supplementary Material online. A description of all basic steps used in our algorithm, including a schematic representation, is available in supplementary Text 2 and figures S2 and S3, Supplementary material online. The associated code is publicly available at https://github.com/nataliamv/SARS-CoV-2-ARTs-Classification.

### Estimating dN/dS

Derived from the global alignment and tree, we estimated dN/dS (*ω*, the ratio between the nonsynonymous substitution rate per nonsynonymous site and the synonymous substitution rate per synonymous site) using the following site, branch, and branch–site models: mixed effects model of evolution (MEME), fixed effects likelihood (FEL), and the fixed effects site-level model (Contrast-FEL) (Kosakovsky Pond and Frost 2005; Kosakovsky Pond et al. 2021; Murrell et al. 2012). For this, the alignment was partitioned into 14 putatively nonrecombinant regions using the genetic algorithm for recombination detection (GARD) (Kosakovsky Pond, Posada, et al. 2006), with all subsequent analyses conducted on the partitioned data. As dN/dS models use the GTR component for the nucleotide evolutionary rate, biased mutation rates are handled. Further, to mitigate the inflation in dN/dS estimates that results from unresolved and/or maladaptive evolution, testing for selection was again restricted to internal nodes/branches of the phylogeny (Kosakovsky Pond, Frost, et al. 2006). Genome-wide comparison of dN/dS estimates across viral genome regions was performed using the Branch-Site Unrestricted Statistical Test for Episodic Diversification (BUSTED) method (Murrell et al. 2015). Finally, the impact of changing biochemical properties at selected sites was further assessed under the Property Informed Models of Evolution method (PRIME) method (HyPhy 2013). Our results were further compared with the selection analysis available for independently sampled SARS-CoV-2 genome data available as of December 2022 (https://observablehq.com/@spond/evolutionary-annotation-of-sars-cov-2-covid-19-genomes-enab) (Kosakovsky Pond).

### Mapping Mutations onto Betacoronavirus Protein Structures

To locate the nonsynonymous mutations identified on viral protein regions of known function, corresponding residues were mapped to available structural data using PyMOL v 2.4.0 (https://pymol.org/2/) (supplementary table S2, Supplementary Material online; see Data Availability section). Mutations were analyzed in the context of their relative proximity to previously reported functional regions, and to each other. N-linked glycosylation sites in S protein sequences were identified by searching for the N-[not P]-[S or T] consensus sequence (Watanabe et al. 2019). None of the mutations identified in this study resulted in generation or deletion of N-linked glycosylation sequons. In parallel, conserved and variable sites identified (including the 30 mutations evidencing homoplasy and/or stepwise evolution across virus species) were mapped onto published protein structures available for the S proteins of the four human-infecting betacoronaviruses studied here (figs. 5 and supplementary fig. S7, Supplementary Material online). Finally, to compare dN/dS distributions between specific domains of the S protein within and across virus species, sites inferred to be under positive or negative selection (PSS, NSS) were mapped onto S protein structures (supplementary Data 2, Supplementary Material online).

### Validation through Resampling and by Comparing Mutational Distributions

To validate our initial observations derived from virus genomes sampled up to May 2021, we sought to determine if the 30 mutations that had been identified initially were also present in the expanded embeco- and sarbecovirus diversity sampled up to July 25th 2022 (corresponding to the final sampling date of this study). Virus diversity now included genome sequences derived from more recently collected human isolates (only made publicly available after our initial sampling), and from other closely related embeco- and sarbecoviruses from nonhuman hosts. The expanded alignment comprises 1,455 sequences (∼700 embecoviruses + SARS-CoV and ∼700 SARS-CoV-2), resulting in 27,503 columns that were realigned under a progressive profile-to-profile approach based on taxonomic relatedness to be further used to estimate an expanded “Maximum Likelihood” tree (following Methods section 2, Supplementary Material online). To additionally explore if the mutations identified were also present in a larger data set representing an expanded SARS-CoV-2 diversity (sampled up to July 25th 2022), a set of 1,400 SARS-CoV-2 genomes denoting “evolutionary successful” virus lineages (supplementary table S3, Supplementary Material online) was examined independently (supplementary Text 3 and fig. S4, Supplementary Material online and 5). Both data sets were analyzed following the steps described in Methods sections 2 and 3, Supplementary Material online, specifically searching for the mutations listed in table 1. Virus taxa included in both resampled data sets are listed in supplementary Data 5, Supplementary Material online. A full description of the sequence subsampling and methodological approach used is available as supplementary Text 3 and figures S4 and S5, Supplementary Material online.

We further sought to explore if the proportion of mutational patterns we classified as putatively denoting homoplasy and/or stepwise evolution were more likely to arise from an evolutionary scenario mostly driven by selection, compared with “random” mutational patterns derived from evolutionary scenarios generally driven by genetic drift. For this purpose, the expanded alignment was translated to amino acid sequences and used to simulate three alignments with “AliSim” (http://www.iqtree.org/doc/AliSim) under the “mimick real alignment” function (mimicking a “real” evolutionary process based on amino acid evolution under a LG model and applied to the inputted original tree). To compare the corresponding proportion of sites scored under homoplasy and/or stepwise evolution, each data set (the expanded and three simulated alignments) was analyzed following the steps described in Methods section 3, Supplementary Material online. The classification of mutational patterns within expanded and simulated data sets also serves the purpose of validating our algorithm, originally developed for analyzing the global data set (that included only OC43, HKU1, SARS-CoV-1, and SARS-CoV-2 sampled from the human host). Associated results and a brief discussion are available as supplementary Text 5, Supplementary Material online.

### Reconstruction of Amino Acid Evolution for Selected Sites

To further confirm our ML-derived results (see Methods section 3, Supplementary Material online), for those mutations displaying cumulative evidence of adaptive convergence (18121, 21623, 21635, and 23948, table 1), we used the expanded data set to infer ancestral states under a Bayesian framework. For each site, we first estimated a MCC (maximum clade credibility) tree from the resampled codon alignment using an SRD06 substitution model (Shapiro et al. 2006) and a strict molecular clock. Coded amino acid traits were then mapped onto the nodes of the MCC tree by performing reconstructions of ancestral states under an asymmetric discrete trait evolution model (DTA) in BEAST v1.8.4 (Lemey et al. 2009; Suchard et al. 2018). The DTA model was run using a Bayesian Skygrid tree prior for 100 × 10^6^ generations and sampled every 10,000 states until all DTA-relevant parameters reached an ESS > 200. For illustrative purposes, figure 3 only shows sites 18121, 21623, and 23948. The amino acid evolution pattern observed for site 21635 is available in supplementary Data 3, Supplementary Material online.

## Supplementary Material

evad076_Supplementary_Data

## Data Availability

Taxa IDs and accession numbers for virus sequences used in this study are provided in the supplementary Data 4 and 5, Supplementary Material online, files. All SARS-CoV-2 genome sequences and associated metadata used in this study are published in GISAID's EpiCoV database under the EPI SET GISAID Identifier: EPI_SET_230131zy. To view the contributors of each individual sequence with details such as the accession number, virus name, collection date, originating, and submitting lab, as well as the list of all authors, visit 10.55876/gis8.230131zy. PBD files used are listed as follows: S protein (HKU1 PDB:5I08, OC43 PDB:6OHW, SARS-CoV-1 PDB:6ACC, and SARS-CoV-2 PDB:6VXX, 6ZGI); Orf1a (SARS-CoV-1 nsp3 PDB:2W2G); and Orf1b (SARS-CoV-2 nsp13 PDB:6XEZ, SARS-CoV-1 nsp14 PDB:5C8S, and SARS-CoV-2 nsp15 PDB:6WLC). The full code for our algorithm is available as open source: https://github.com/nataliamv/SARS-CoV-2-ARTs-Classification. An interactive notebook with our full selection analysis results is available at https://observablehq.com/@spond/beta-cov-analysis.
