# Supplementary material for: Identification of Evolutionary Trajectories Shared across Human Betacoronaviruses
Source: Genome Biol Evol. 2023 May 23;15(6):evad076. doi: 10.1093/gbe/evad076 (PMC10282123; doi:10.1093/gbe/evad076)
Supplement: evad076_Supplementary_Data [file evad076_supplementary_data.zip › GISAID_Supplemental_Table_230131zy.pdf]

SUPPLEMENTAL TABLE

Data Availability

GISAID Identifier:	EPI_SET_230131zy
doi:	10.55876/gis8.230131zy

All genome sequences and associated metadata in this dataset are published in GISAID's EpiCoV database. To view the contributors of each individual sequence with details such as accession number, Virus name, Collection date, Originating Lab and Submitting Lab and the list of Authors, visit 10.55876/gis8.230131zy

Data Snapshot

- EPI_SET_230131zy is composed of 1,396 individual genome sequences.
- The collection dates range from 2019-12-24 to 2022-07-13;
- Data were collected in 78 countries and territories;
- All sequences in this dataset are compared relative to hCoV-19/Wuhan/WIV04/2019 (WIV04), the official reference sequence employed by GISAID (EPI_ISL_402124). Learn more at https://gisaid.org/WIV04.
