# Supplementary material for: Identification of Evolutionary Trajectories Shared across Human Betacoronaviruses
Source: Genome Biol Evol. 2023 May 23;15(6):evad076. doi: 10.1093/gbe/evad076 (PMC10282123; doi:10.1093/gbe/evad076)
Supplement: evad076_Supplementary_Data [file evad076_supplementary_data.zip › Supplementary_Data_1_ML_treefiles.pdf]

RAxML tree Orf1ab+Spike, 100 bootstraps

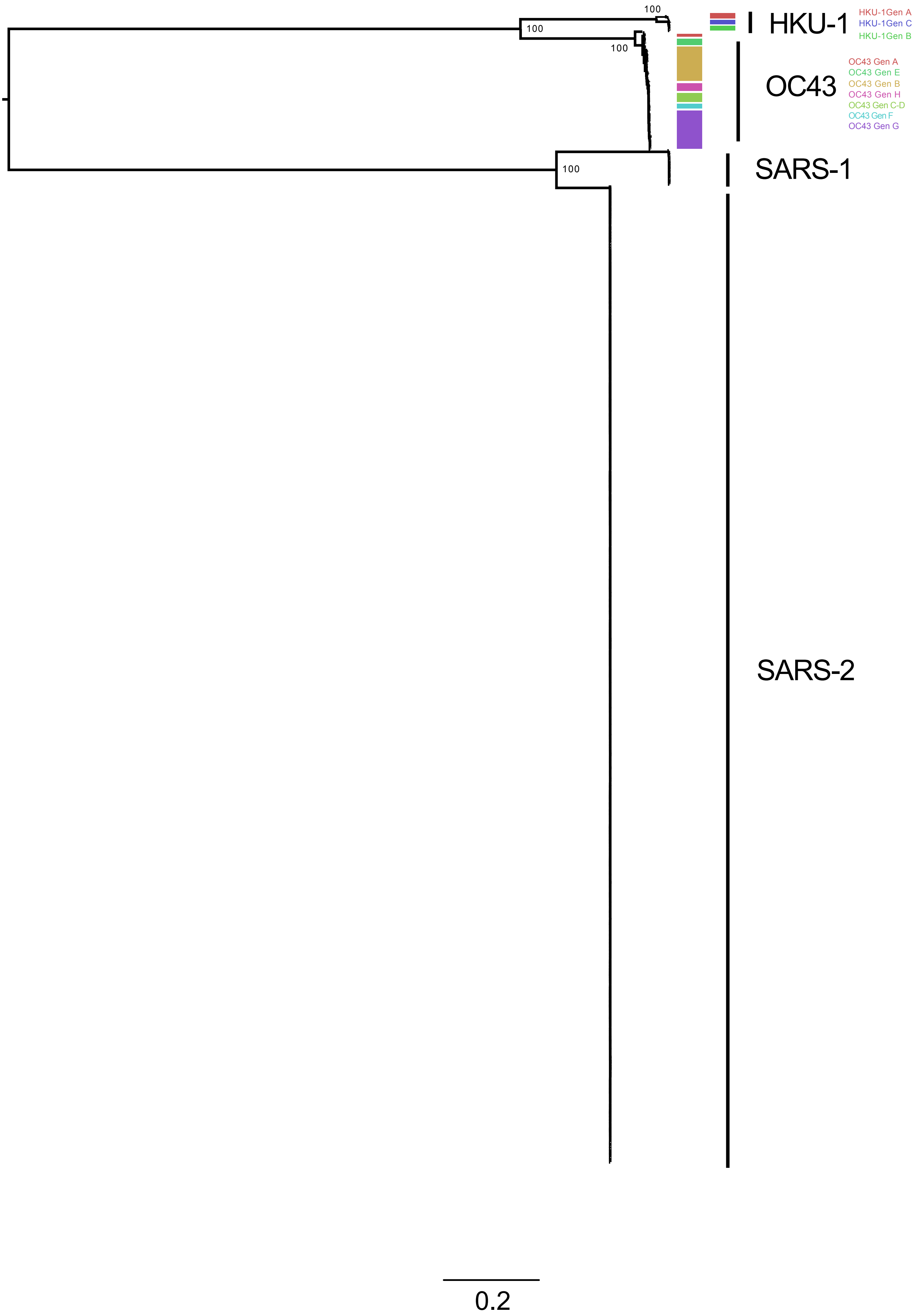

RAxML tree Orf1a, 100 bootstraps

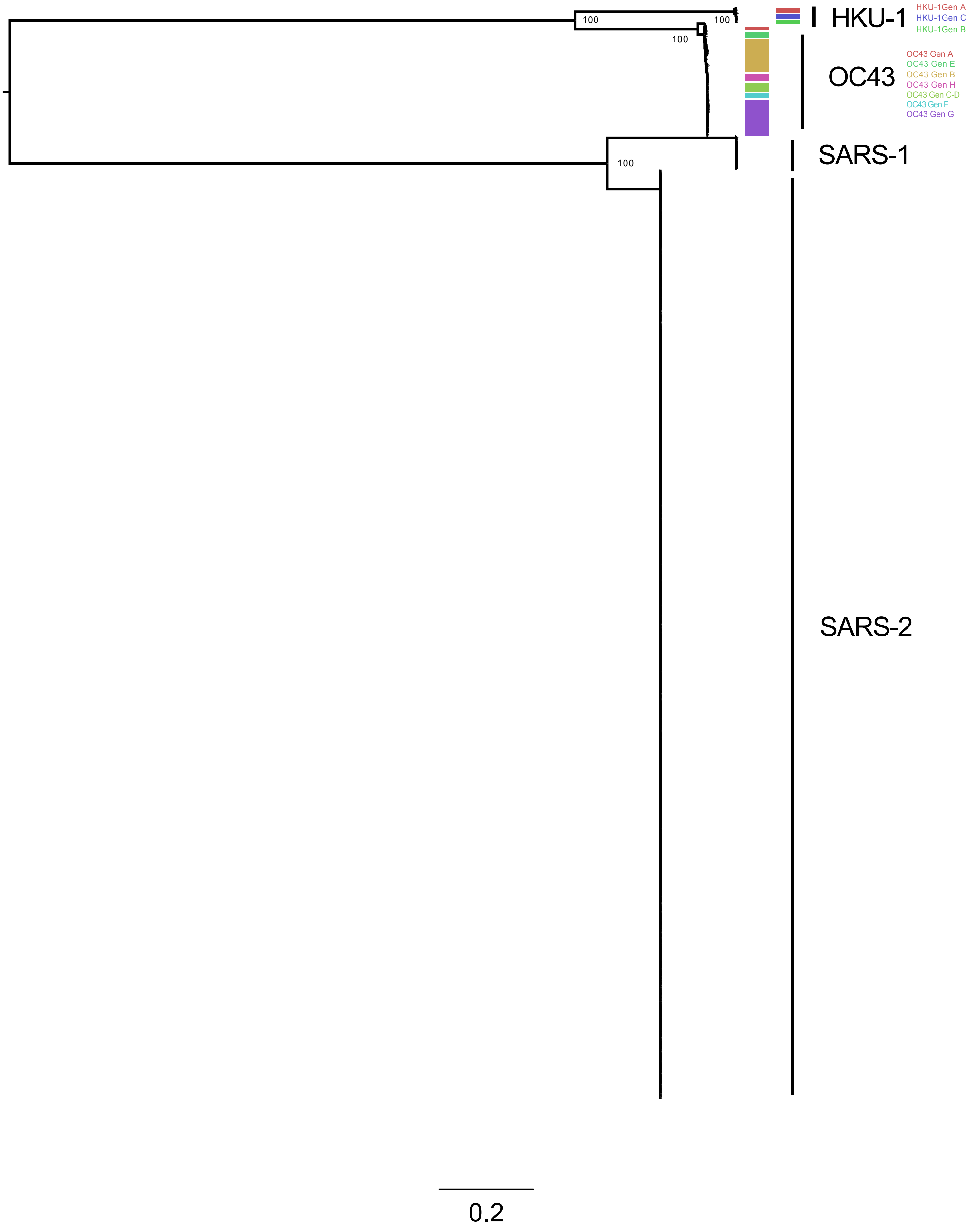

RAxML tree Orf1b, 100 bootstraps

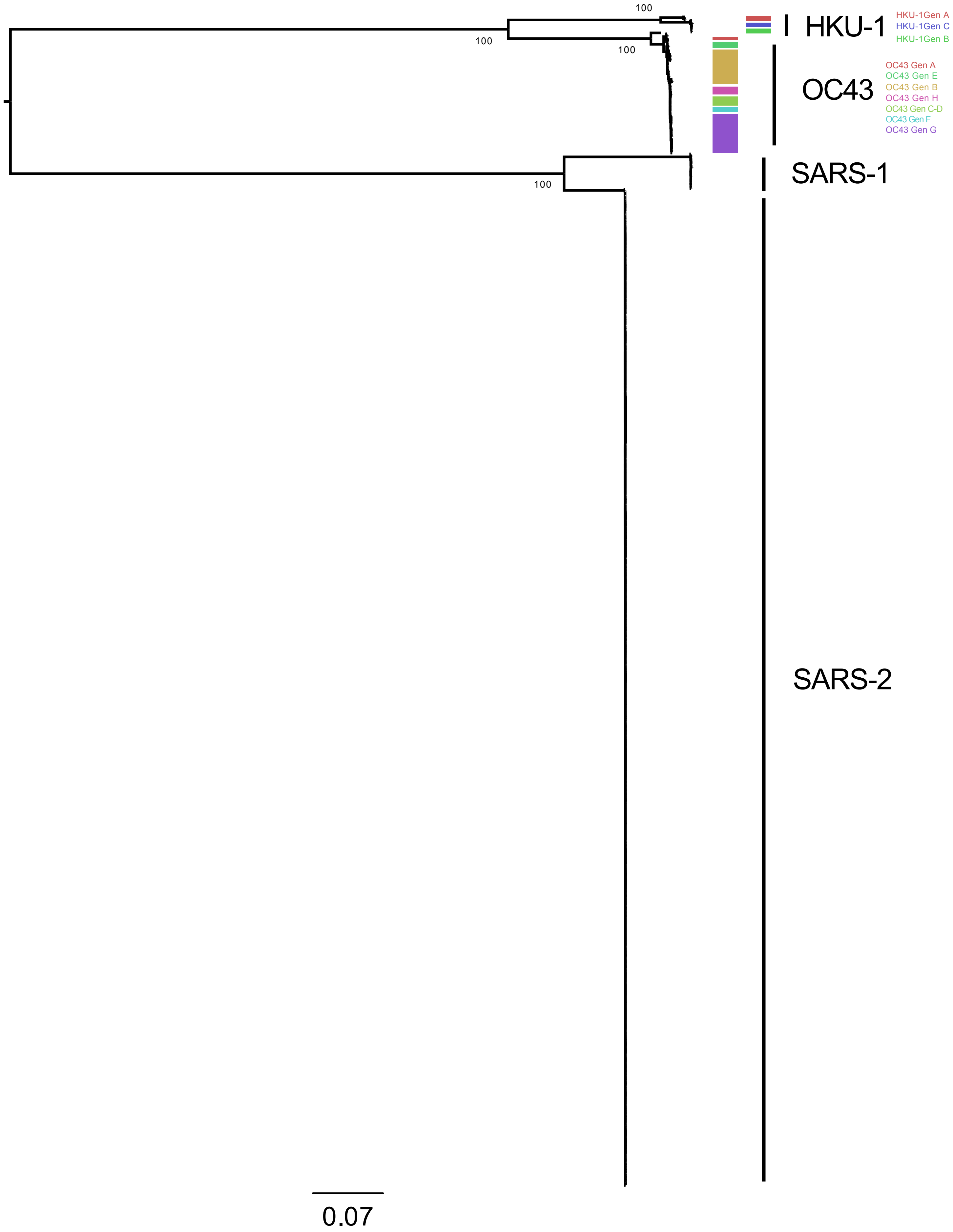

RAxML tree Spike, 100 bootstraps

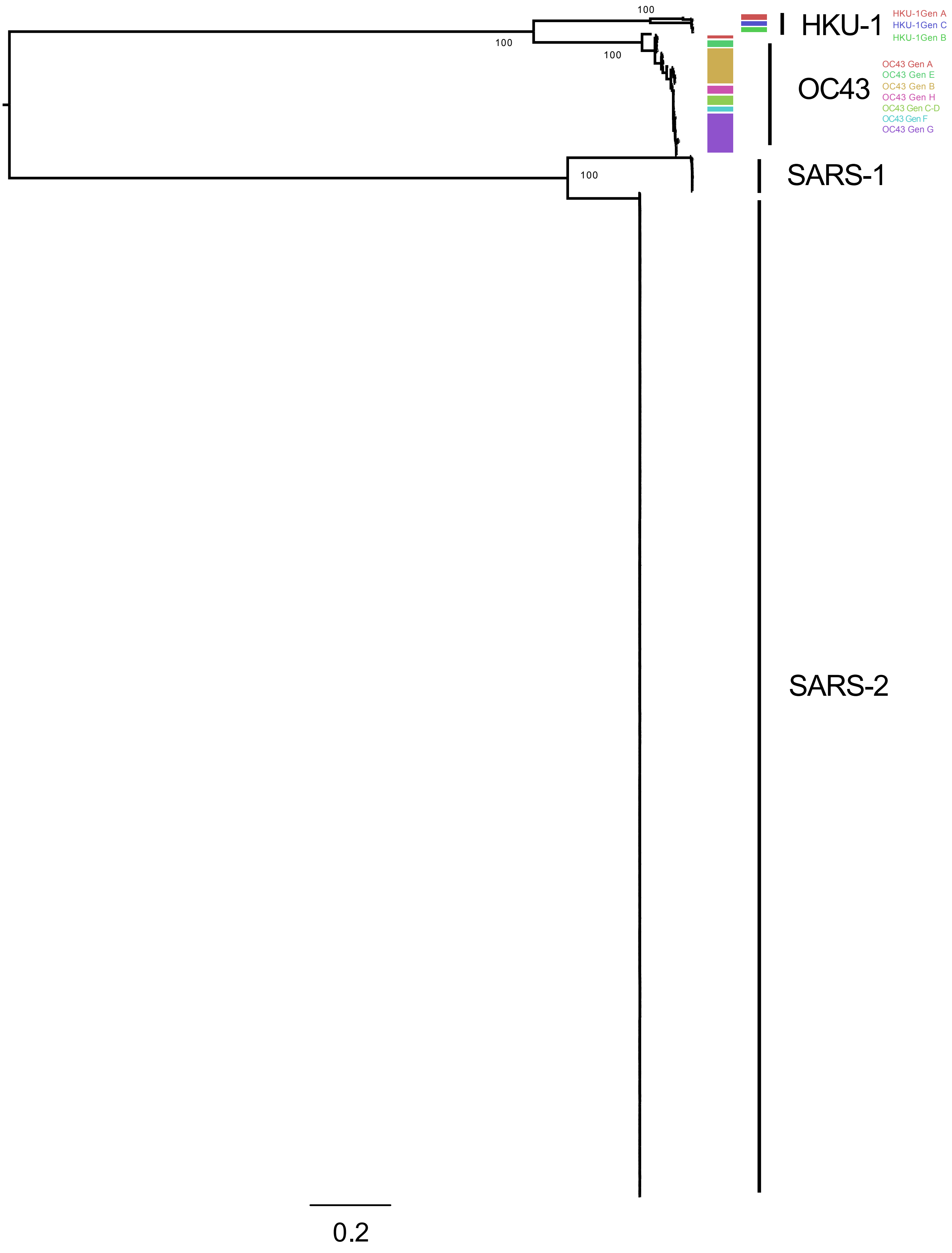

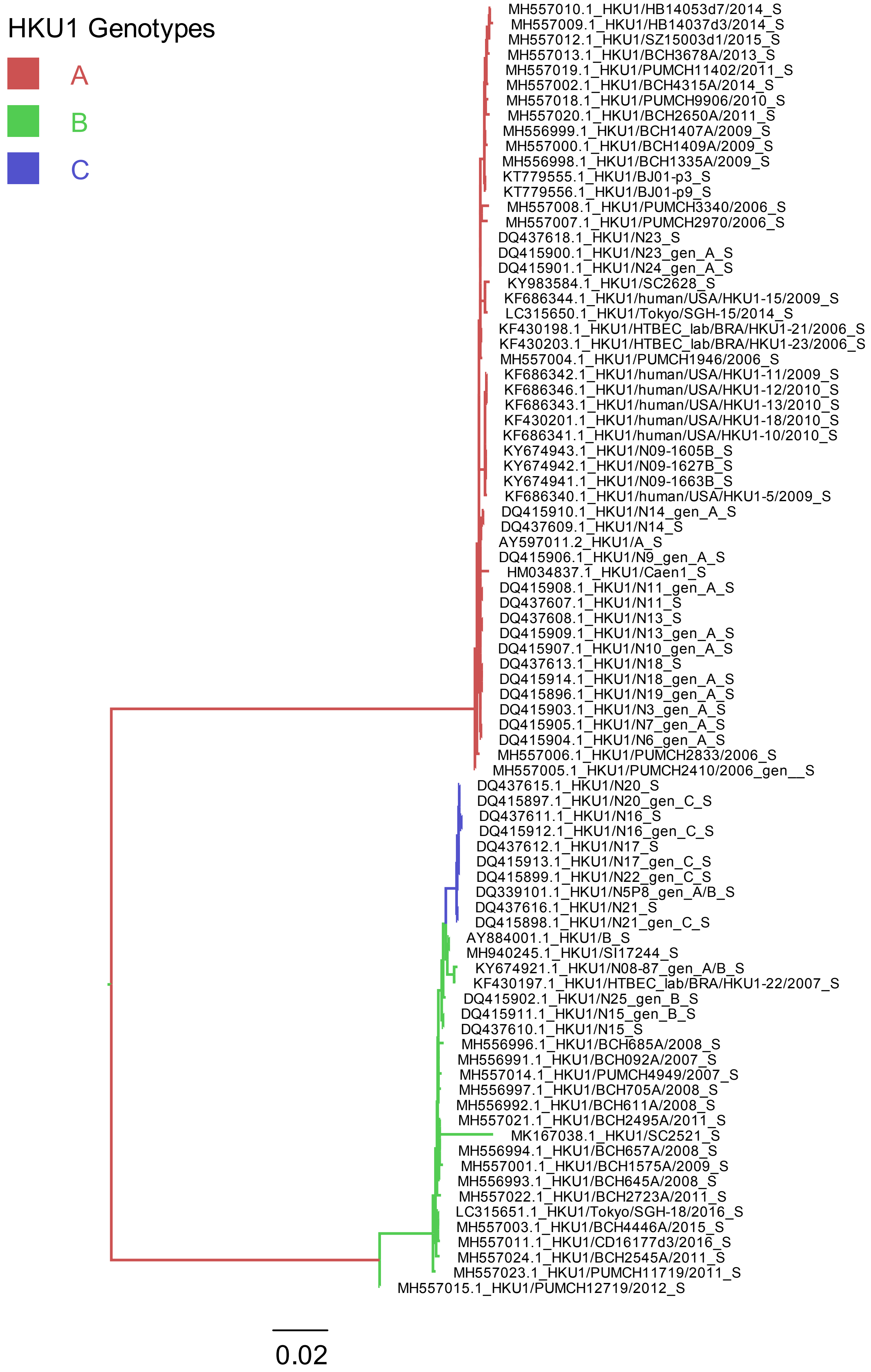
HKU1 Genotypes
A
B
C
MH557010.1_HKU1/HB14053d7/2014_S
MH557009.1_HKU1/HB14037d3/2014_S
MH557012.1_HKU1/SZ15003d1/2015_S
MH557013.1_HKU1/BCH3678A/2013_S
MH557019.1_HKU1/PUMCH11402/2011_S
MH557002.1_HKU1/BCH4315A/2014_S
MH557018.1_HKU1/PUMCH9906/2010_S
MH557020.1_HKU1/BCH2650A/2011_S
MH556999.1_HKU1/BCH1407A/2009_S
MH557000.1_HKU1/BCH1409A/2009_S
MH556998.1_HKU1/BCH1335A/2009_S
KT779555.1_HKU1/BJ01-p3_S
KT779556.1_HKU1/BJ01-p9_S
MH557008.1_HKU1/PUMCH3340/2006_S
MH557007.1_HKU1/PUMCH2970/2006_S
DQ437618.1_HKU1/N23_S
DQ415900.1_HKU1/N23_gen_A_S
DQ415901.1_HKU1/N24_gen_A_S
KY983584.1_HKU1/SC2628_S
KF686344.1_HKU1/human/USA/HKU1-15/2009_S
LC315650.1_HKU1/Tokyo/SGH-15/2014_S
KF430198.1_HKU1/HTBEC_lab/BRA/HKU1-21/2006_S
KF430203.1_HKU1/HTBEC_lab/BRA/HKU1-23/2006_S
MH557004.1_HKU1/PUMCH1946/2006_S
KF686342.1_HKU1/human/USA/HKU1-11/2009_S
KF686346.1_HKU1/human/USA/HKU1-12/2010_S
KF686343.1_HKU1/human/USA/HKU1-13/2010_S
KF430201.1_HKU1/human/USA/HKU1-18/2010_S
KF686341.1_HKU1/human/USA/HKU1-10/2010_S
KY674943.1_HKU1/N09-1605B_S
KY674942.1_HKU1/N09-1627B_S
KY674941.1_HKU1/N09-1663B_S
KF686340.1_HKU1/human/USA/HKU1-5/2009_S
DQ415910.1_HKU1/N14_gen_A_S
DQ437609.1_HKU1/N14_S
AY597011.2_HKU1/A_S
DQ415906.1_HKU1/N9_gen_A_S
HM034837.1_HKU1/Caen1_S
DQ415908.1_HKU1/N11_gen_A_S
DQ437607.1_HKU1/N11_S
DQ437608.1_HKU1/N13_S
DQ415909.1_HKU1/N13_gen_A_S
DQ415907.1_HKU1/N10_gen_A_S
DQ437613.1_HKU1/N18_S
DQ415914.1_HKU1/N18_gen_A_S
DQ415896.1_HKU1/N19_gen_A_S
DQ415903.1_HKU1/N3_gen_A_S
DQ415905.1_HKU1/N7_gen_A_S
DQ415904.1_HKU1/N6_gen_A_S
MH557006.1_HKU1/PUMCH2833/2006_S
MH557005.1_HKU1/PUMCH2410/2006_gen__S
DQ437615.1_HKU1/N20_S
DQ415897.1_HKU1/N20_gen_C_S
DQ437611.1_HKU1/N16_S
DQ415912.1_HKU1/N16_gen_C_S
DQ437612.1_HKU1/N17_S
DQ415913.1_HKU1/N17_gen_C_S
DQ415899.1_HKU1/N22_gen_C_S
DQ339101.1_HKU1/N5P8_gen_A/B_S
DQ437616.1_HKU1/N21_S
DQ415898.1_HKU1/N21_gen_C_S
AY884001.1_HKU1/B_S
MH940245.1_HKU1/SI17244_S
KY674921.1_HKU1/N08-87_gen_A/B_S
KF430197.1_HKU1/HTBEC_lab/BRA/HKU1-22/2007_S
DQ415902.1_HKU1/N25_gen_B_S
DQ415911.1_HKU1/N15_gen_B_S
DQ437610.1_HKU1/N15_S
MH556996.1_HKU1/BCH685A/2008_S
MH556991.1_HKU1/BCH092A/2007_S
MH557014.1_HKU1/PUMCH4949/2007_S
MH556997.1_HKU1/BCH705A/2008_S
MH556992.1_HKU1/BCH611A/2008_S
MH557021.1_HKU1/BCH2495A/2011_S
MK167038.1_HKU1/SC2521_S
MH556994.1_HKU1/BCH657A/2008_S
MH557001.1_HKU1/BCH1575A/2009_S
MH556993.1_HKU1/BCH645A/2008_S
MH557022.1_HKU1/BCH2723A/2011_S
LC315651.1_HKU1/Tokyo/SGH-18/2016_S
MH557003.1_HKU1/BCH4446A/2015_S
MH557011.1_HKU1/CD16177d3/2016_S
MH557024.1_HKU1/BCH2545A/2011_S
MH557023.1_HKU1/PUMCH11719/2011_S
MH557015.1_HKU1/PUMCH12719/2012_S
0.02

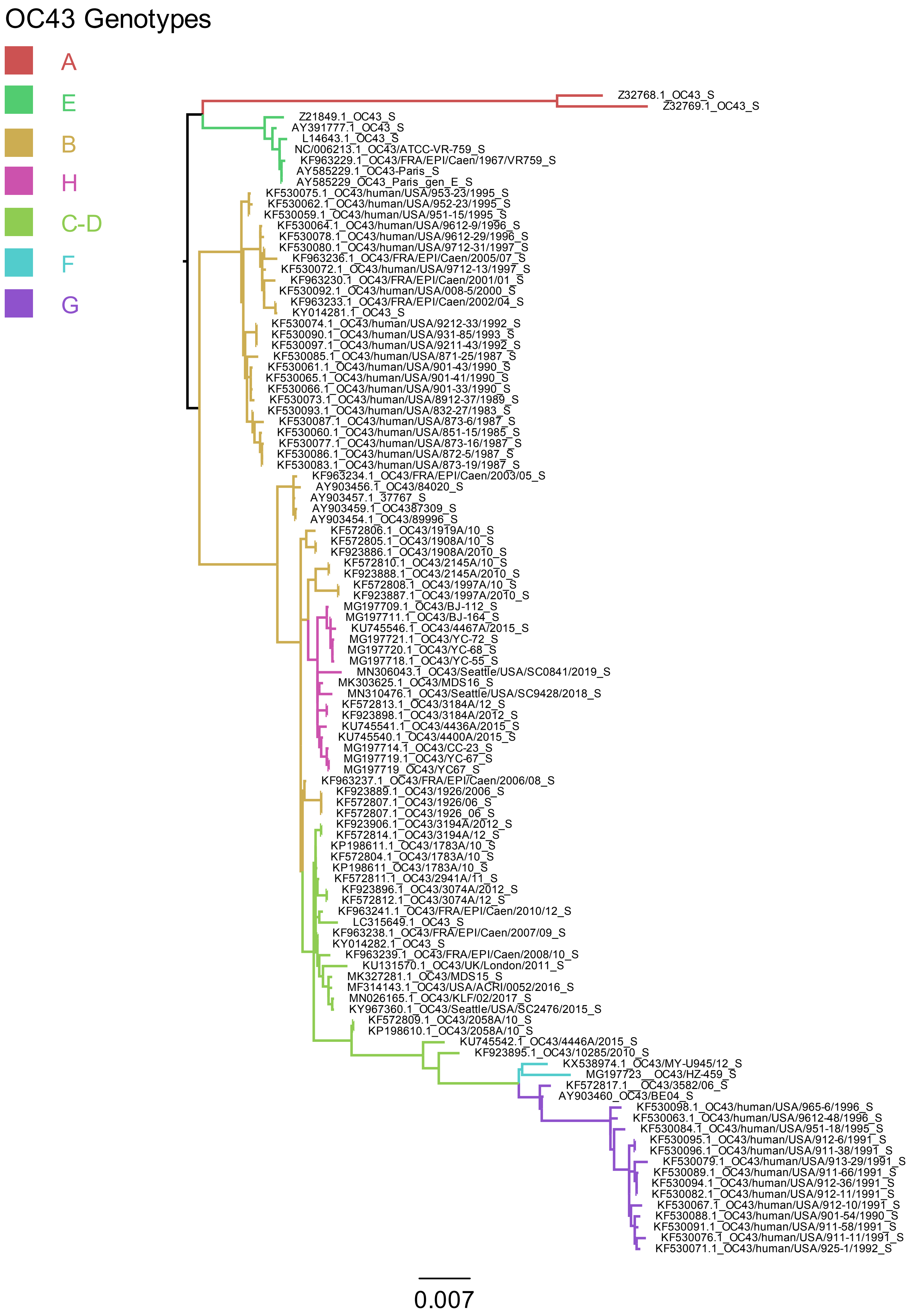
OC43 Genotypes
A
E
B
H
C-D
F
G
Z32768.1_OC43_S
Z32769.1_OC43_S
Z21849.1_OC43_S
AY391777.1_OC43_S
L14643.1_OC43_S
NC/006213.1_OC43/ATCC-VR-759_S
KF963229.1_OC43/FRA/EPI/Caen/1967/VR759_S
AY585229.1_OC43-Paris_S
AY585229_OC43_Paris_gen_E_S
KF530075.1_OC43/human/USA/953-23/1995_S
KF530062.1_OC43/human/USA/952-23/1995_S
KF530059.1_OC43/human/USA/951-15/1995_S
KF530064.1_OC43/human/USA/9612-9/1996_S
KF530078.1_OC43/human/USA/9612-29/1996_S
KF530080.1_OC43/human/USA/9712-31/1997_S
KF963236.1_OC43/FRA/EPI/Caen/2005/07_S
KF530072.1_OC43/human/USA/9712-13/1997_S
KF963230.1_OC43/FRA/EPI/Caen/2001/01_S
KF530092.1_OC43/human/USA/008-5/2000_S
KF963233.1_OC43/FRA/EPI/Caen/2002/04_S
KY014281.1_OC43_S
KF530074.1_OC43/human/USA/9212-33/1992_S
KF530090.1_OC43/human/USA/931-85/1993_S
KF530097.1_OC43/human/USA/9211-43/1992_S
KF530085.1_OC43/human/USA/871-25/1987_S
KF530061.1_OC43/human/USA/901-43/1990_S
KF530065.1_OC43/human/USA/901-41/1990_S
KF530066.1_OC43/human/USA/901-33/1990_S
KF530073.1_OC43/human/USA/8912-37/1989_S
KF530093.1_OC43/human/USA/832-27/1983_S
KF530087.1_OC43/human/USA/873-6/1987_S
KF530060.1_OC43/human/USA/851-15/1985_S
KF530077.1_OC43/human/USA/873-16/1987_S
KF530086.1_OC43/human/USA/872-5/1987_S
KF530083.1_OC43/human/USA/873-19/1987_S
KF963234.1_OC43/FRA/EPI/Caen/2003/05_S
AY903456.1_OC43/84020_S
AY903457.1_37767_S
AY903459.1_OC4387309_S
AY903454.1_OC43/89996_S
KF572806.1_OC43/1919A/10_S
KF572805.1_OC43/1908A/10_S
KF923886.1_OC43/1908A/2010_S
KF572810.1_OC43/2145A/10_S
KF923888.1_OC43/2145A/2010_S
KF572808.1_OC43/1997A/10_S
KF923887.1_OC43/1997A/2010_S
MG197709.1_OC43/BJ-112_S
MG197711.1_OC43/BJ-164_S
KU745546.1_OC43/4467A/2015_S
MG197721.1_OC43/YC-72_S
MG197720.1_OC43/YC-68_S
MG197718.1_OC43/YC-55_S
MN306043.1_OC43/Seattle/USA/SC0841/2019_S
MK303625.1_OC43/MDS16_S
MN310476.1_OC43/Seattle/USA/SC9428/2018_S
KF572813.1_OC43/3184A/12_S
KF923898.1_OC43/3184A/2012_S
KU745541.1_OC43/4436A/2015_S
KU745540.1_OC43/4400A/2015_S
MG197714.1_OC43/CC-23_S
MG197719.1_OC43/YC-67_S
MG197719_OC43/YC67_S
KF963237.1_OC43/FRA/EPI/Caen/2006/08_S
KF923889.1_OC43/1926/2006_S
KF572807.1_OC43/1926/06_S
KF572807.1_OC43/1926_06_S
KF923906.1_OC43/3194A/2012_S
KF572814.1_OC43/3194A/12_S
KP198611.1_OC43/1783A/10_S
KF572804.1_OC43/1783A/10_S
KP198611_OC43/1783A/10_S
KF572811.1_OC43/2941A/11_S
KF923896.1_OC43/3074A/2012_S
KF572812.1_OC43/3074A/12_S
KF963241.1_OC43/FRA/EPI/Caen/2010/12_S
LC315649.1_OC43_S
KF963238.1_OC43/FRA/EPI/Caen/2007/09_S
KY014282.1_OC43_S
KF963239.1_OC43/FRA/EPI/Caen/2008/10_S
KU131570.1_OC43/UK/London/2011_S
MK327281.1_OC43/MDS15_S
MF314143.1_OC43/USA/ACRI/0052/2016_S
MN026165.1_OC43/KLF/02/2017_S
KY967360.1_OC43/Seattle/USA/SC2476/2015_S
KF572809.1_OC43/2058A/10_S
KP198610.1_OC43/2058A/10_S
KU745542.1_OC43/4446A/2015_S
KF923895.1_OC43/10285/2010_S
KX538974.1_OC43/MY-U945/12_S
MG197723__OC43/HZ-459_S
KF572817.1__OC43/3582/06_S
AY903460_OC43/BE04_S
KF530098.1_OC43/human/USA/965-6/1996_S
KF530063.1_OC43/human/USA/9612-48/1996_S
KF530084.1_OC43/human/USA/951-18/1995_S
KF530095.1_OC43/human/USA/912-6/1991_S
KF530096.1_OC43/human/USA/911-38/1991_S
KF530079.1_OC43/human/USA/913-29/1991_S
KF530089.1_OC43/human/USA/911-66/1991_S
KF530094.1_OC43/human/USA/912-36/1991_S
KF530082.1_OC43/human/USA/912-11/1991_S
KF530067.1_OC43/human/USA/912-10/1991_S
KF530088.1_OC43/human/USA/901-54/1990_S
KF530091.1_OC43/human/USA/911-58/1991_S
KF530076.1_OC43/human/USA/911-11/1991_S
KF530071.1_OC43/human/USA/925-1/1992_S
0.007
