# Supplementary material for: Identification of Evolutionary Trajectories Shared across Human Betacoronaviruses
Source: Genome Biol Evol. 2023 May 23;15(6):evad076. doi: 10.1093/gbe/evad076 (PMC10282123; doi:10.1093/gbe/evad076)
Supplement: evad076_Supplementary_Data [file evad076_supplementary_data.zip › Supplementary_Data_3_RASML_Orf1ab_S.pdf]

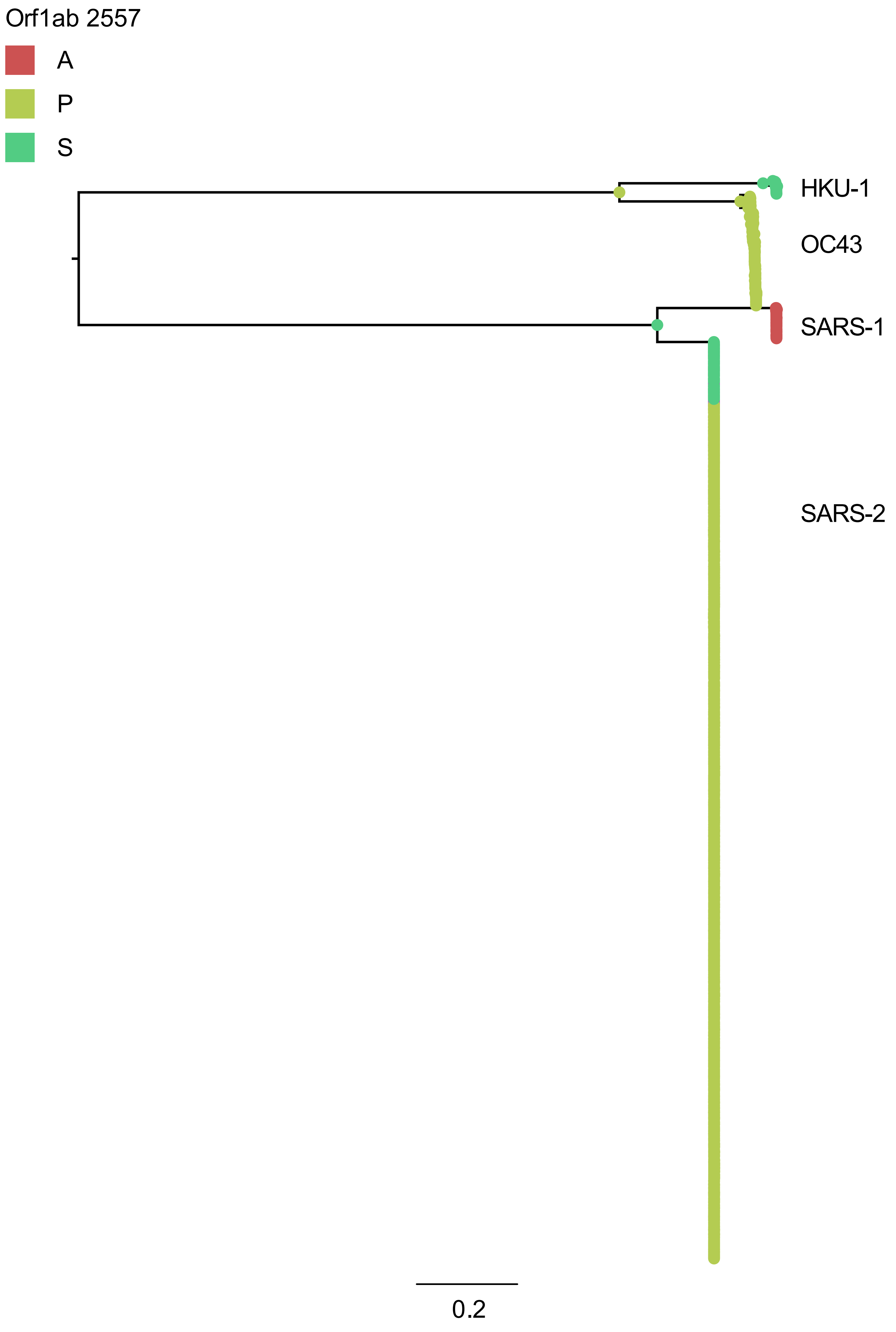
Orf1ab 2557
A
P
S
HKU-1
OC43
SARS-1
SARS-2
0.2

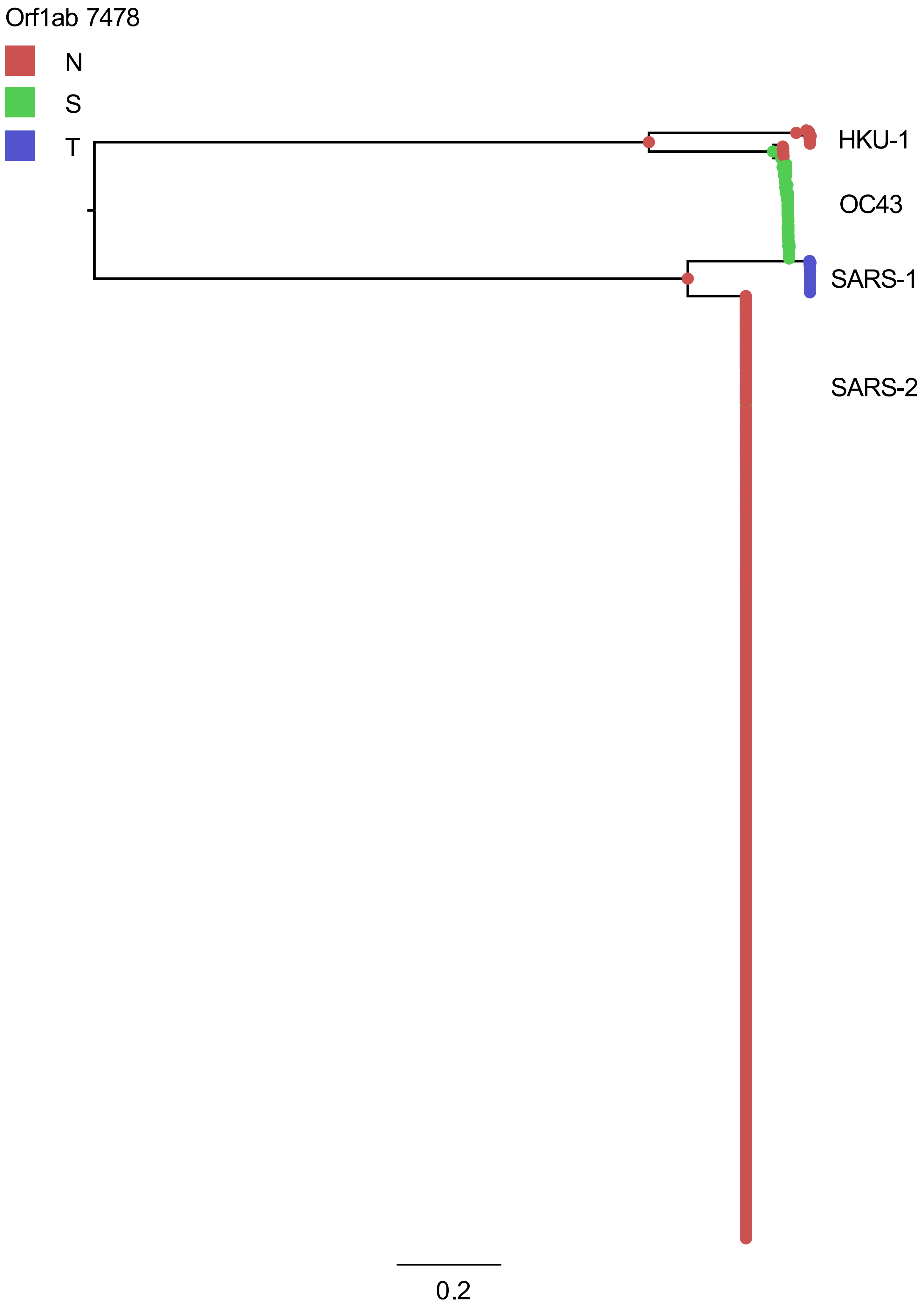
Orf1ab 7478
N
S
T
HKU-1
OC43
SARS-1
SARS-2
0.2

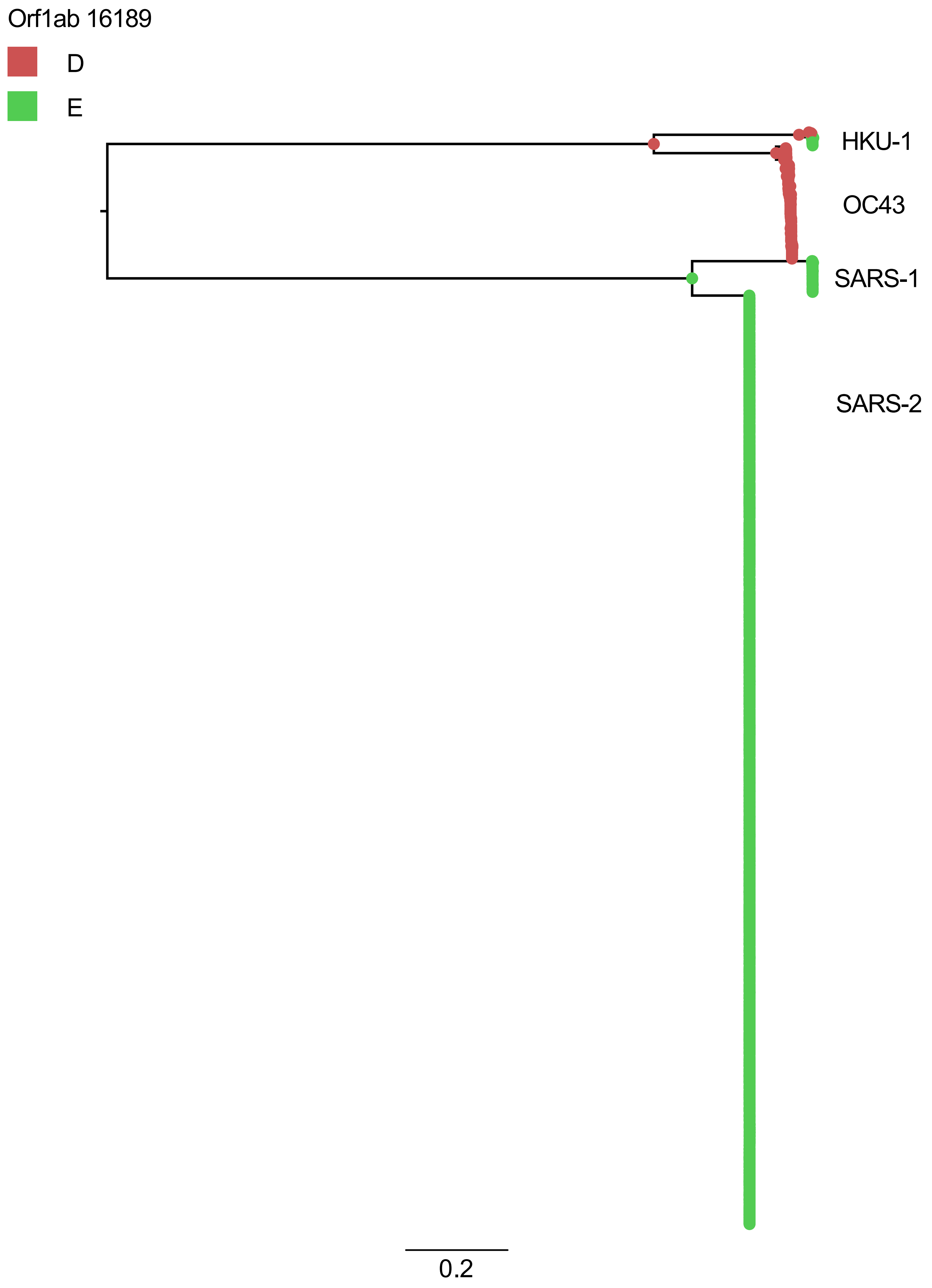
Orf1ab 16189
D
E
HKU-1
OC43
SARS-1
SARS-2
0.2

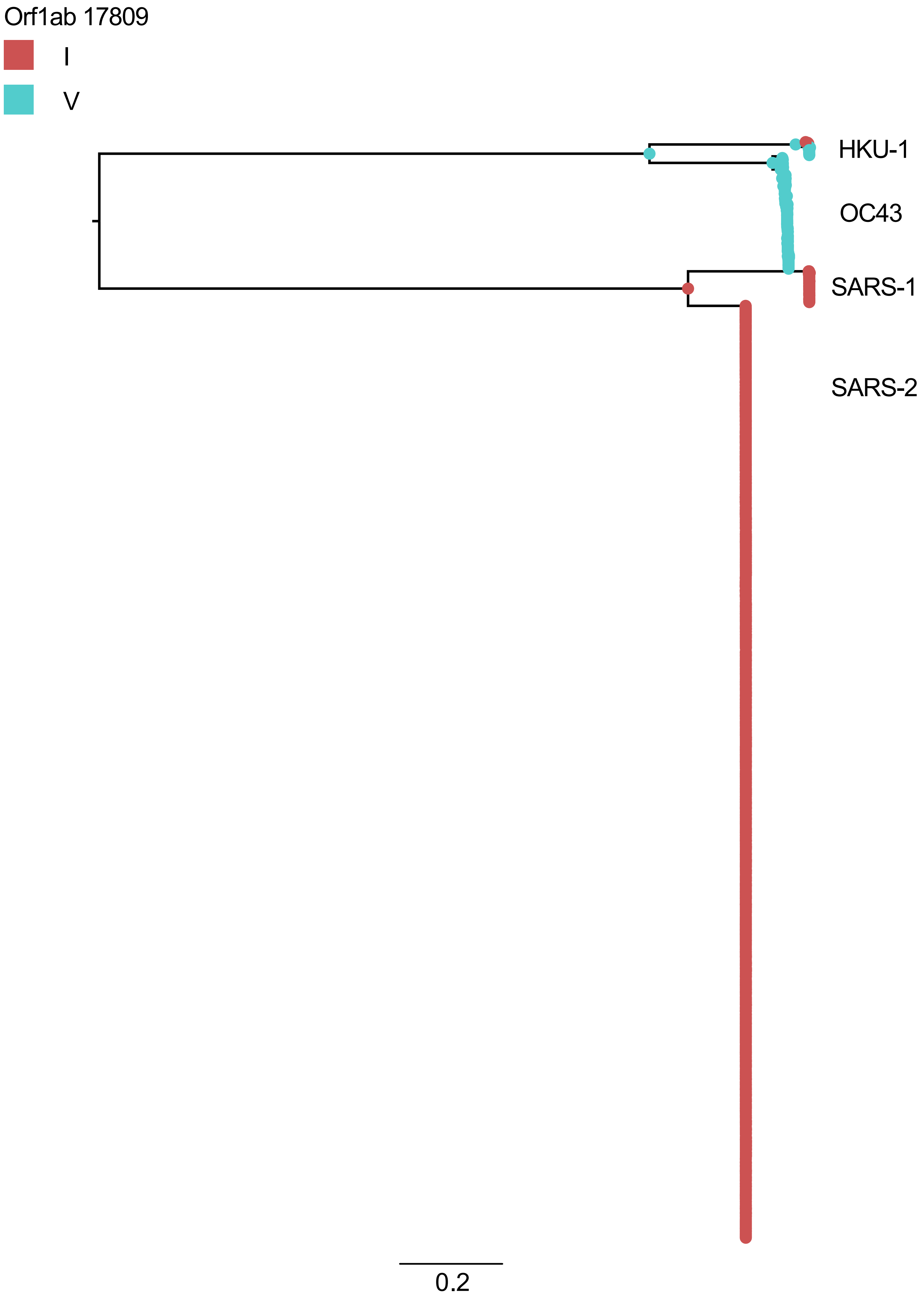
Orf1ab 17809
I
V
HKU-1
OC43
SARS-1
SARS-2
0.2

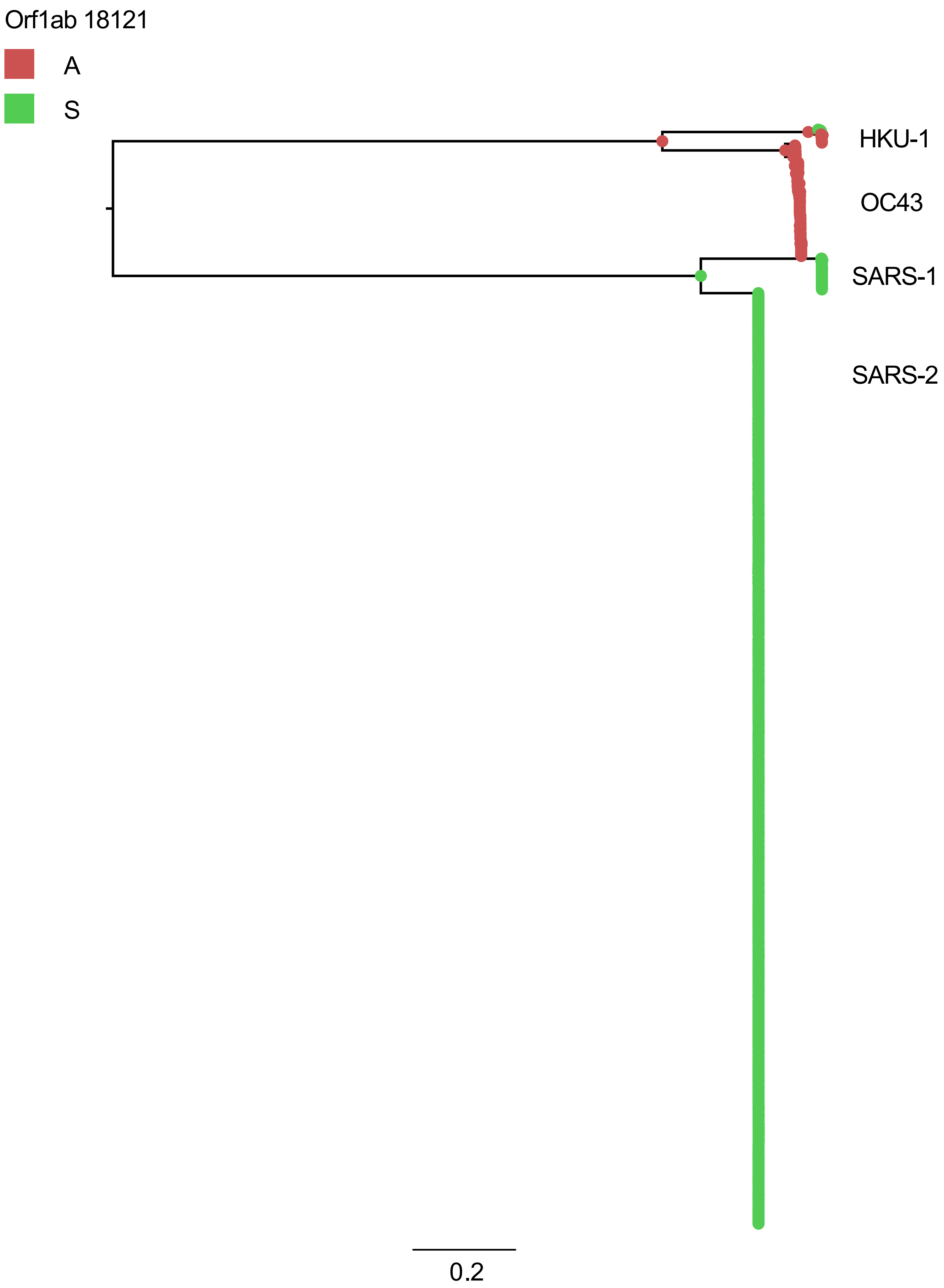

Orf1ab 18121
A
S
HKU-1
OC43
SARS-1
SARS-2
0.2

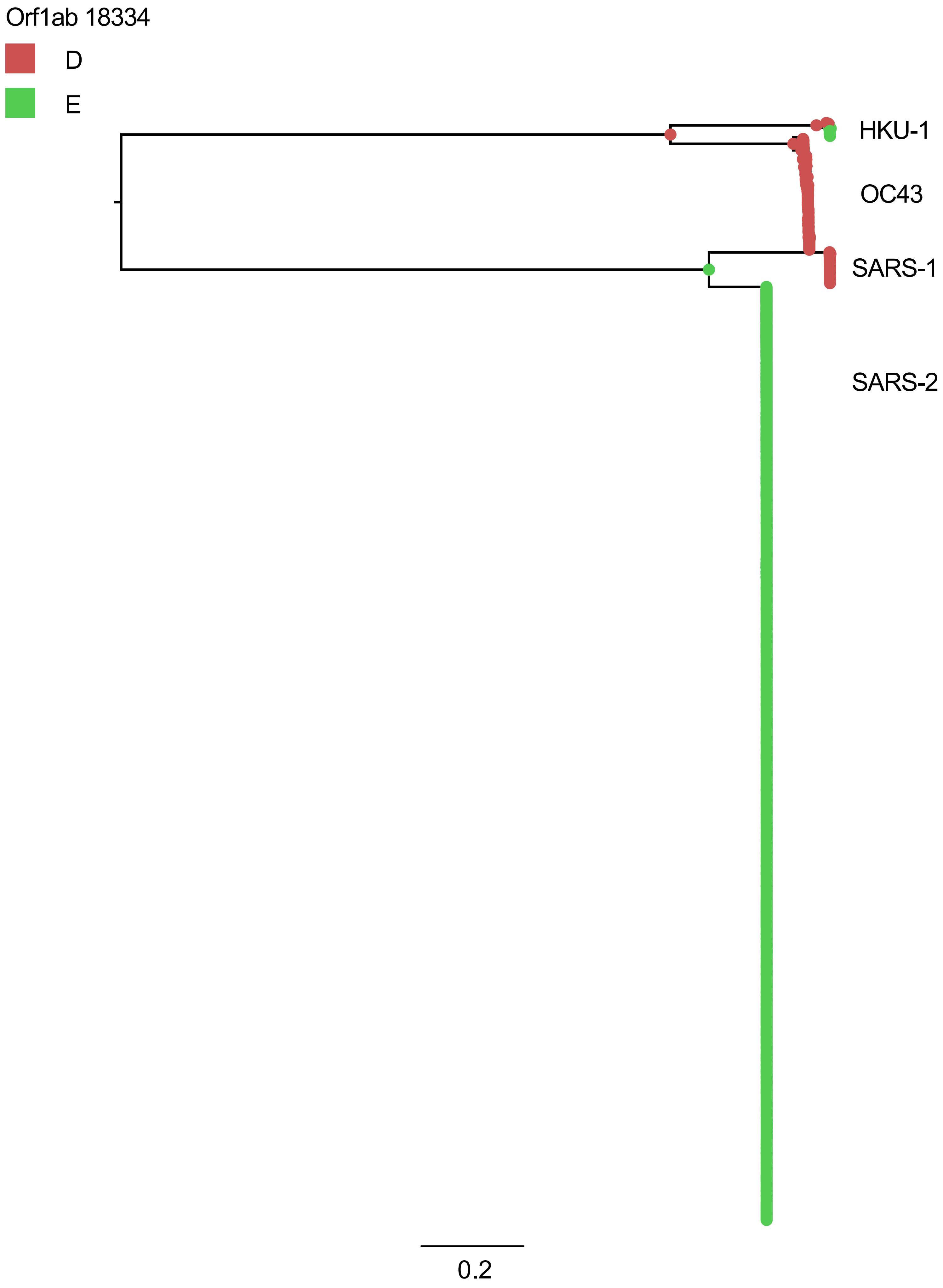

Orf1ab 18334
D
E
HKU-1
OC43
SARS-1
SARS-2
0.2

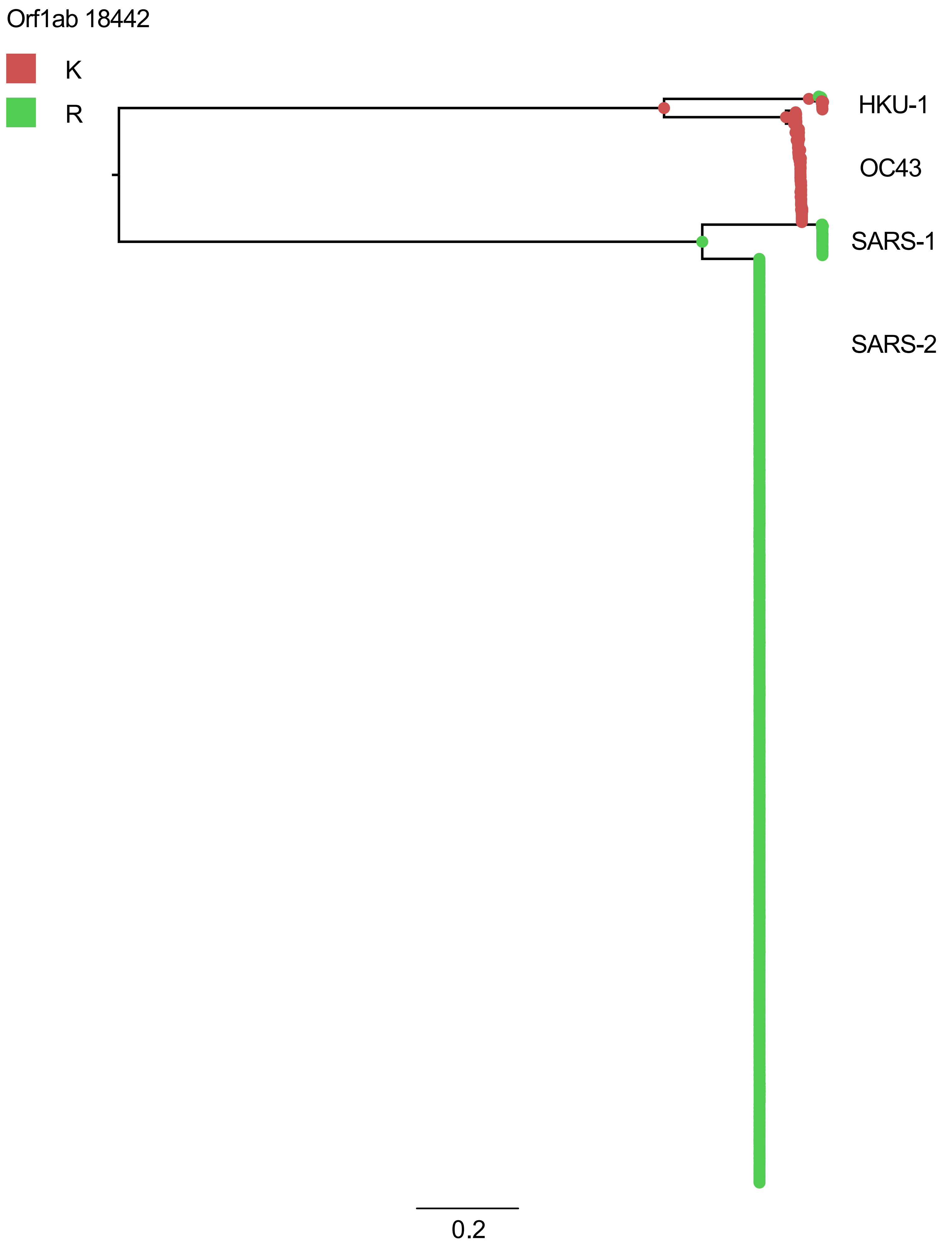
Orf1ab 18442
K
R
HKU-1
OC43
SARS-1
SARS-2
0.2

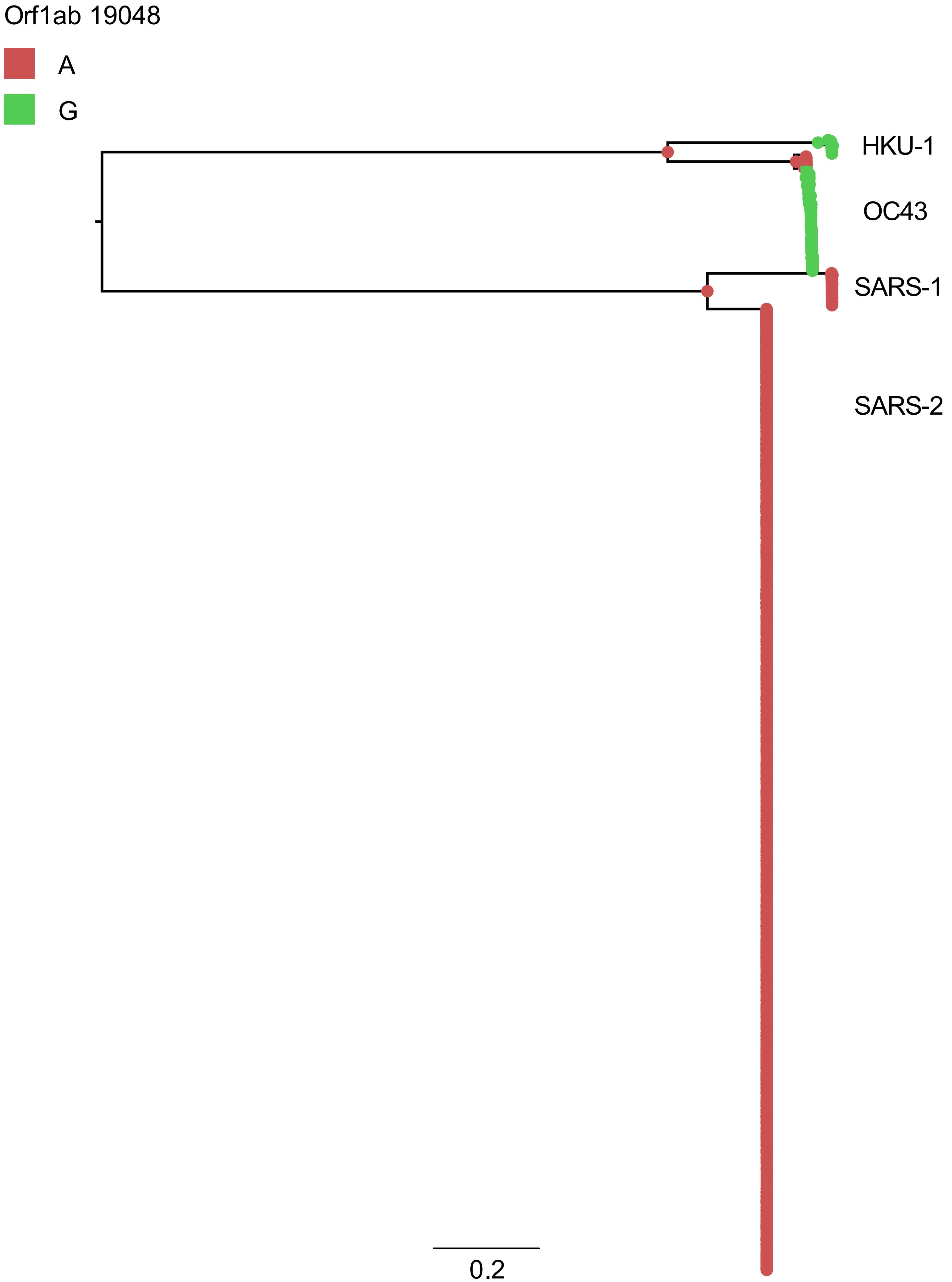
Orf1ab 19048
A
G
HKU-1
OC43
SARS-1
SARS-2
0.2

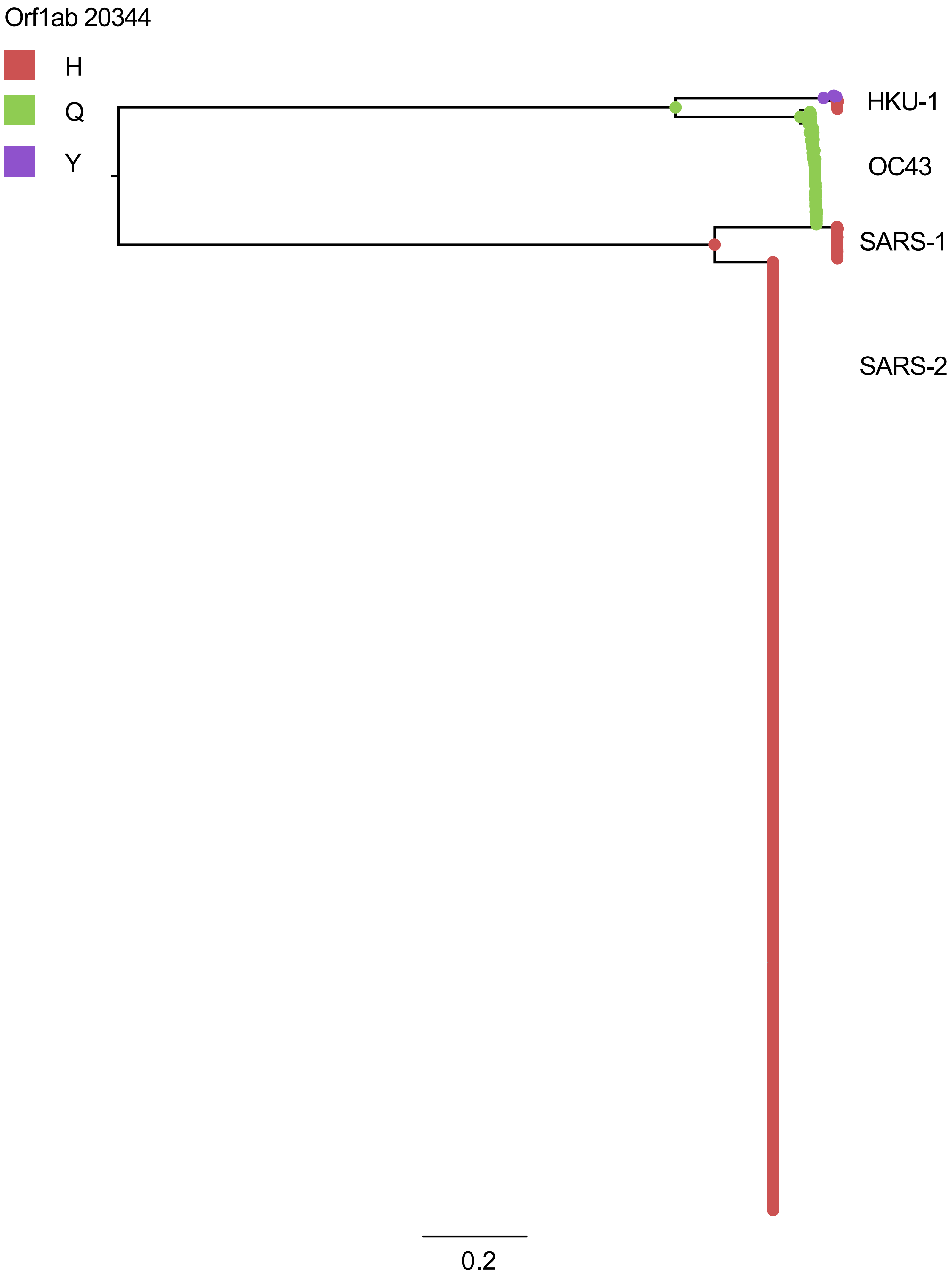

Orf1ab 20344
H
Q
Y
HKU-1
OC43
SARS-1
SARS-2
0.2

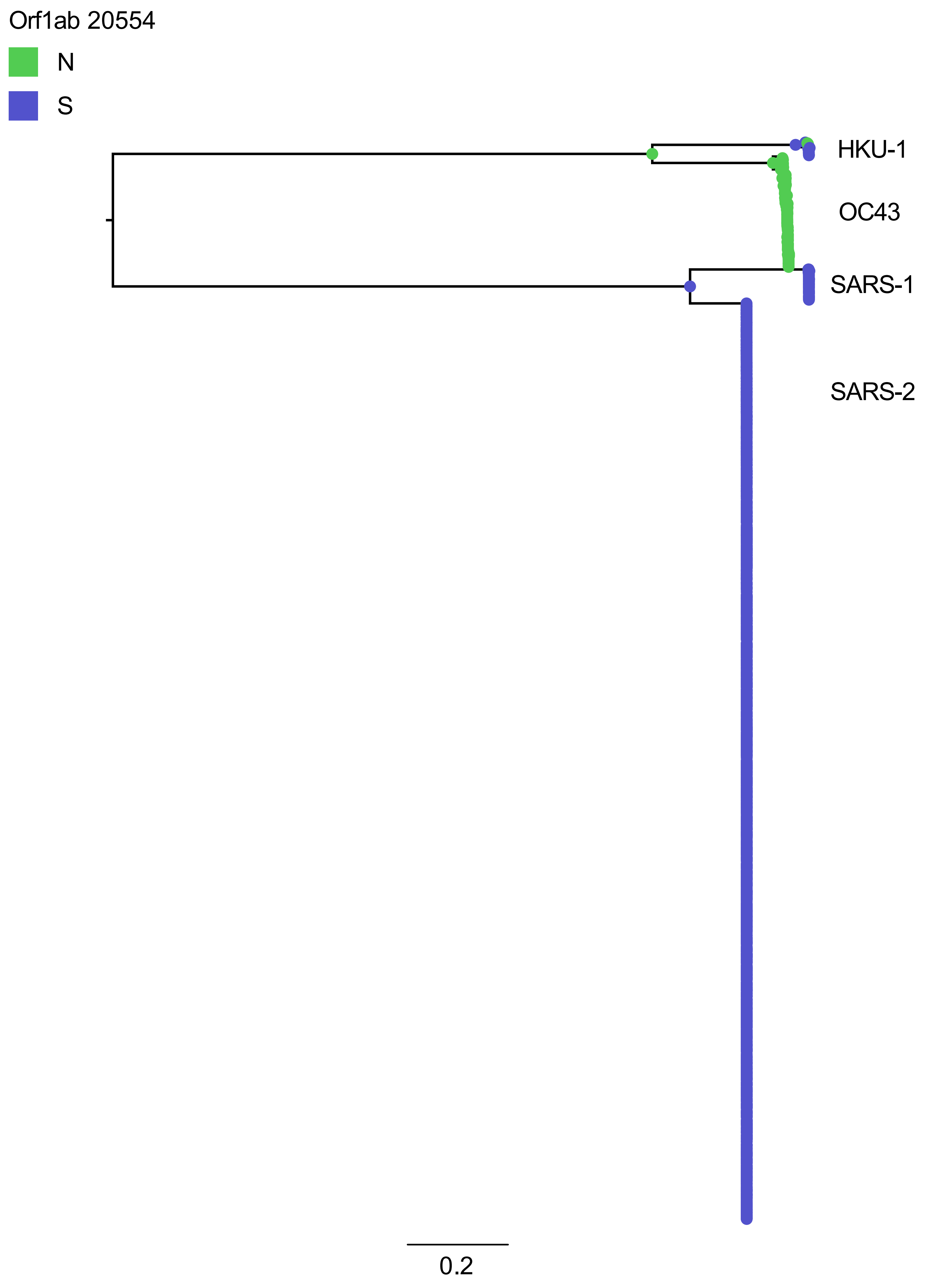
Orf1ab 20554
N
S
HKU-1
OC43
SARS-1
SARS-2
0.2

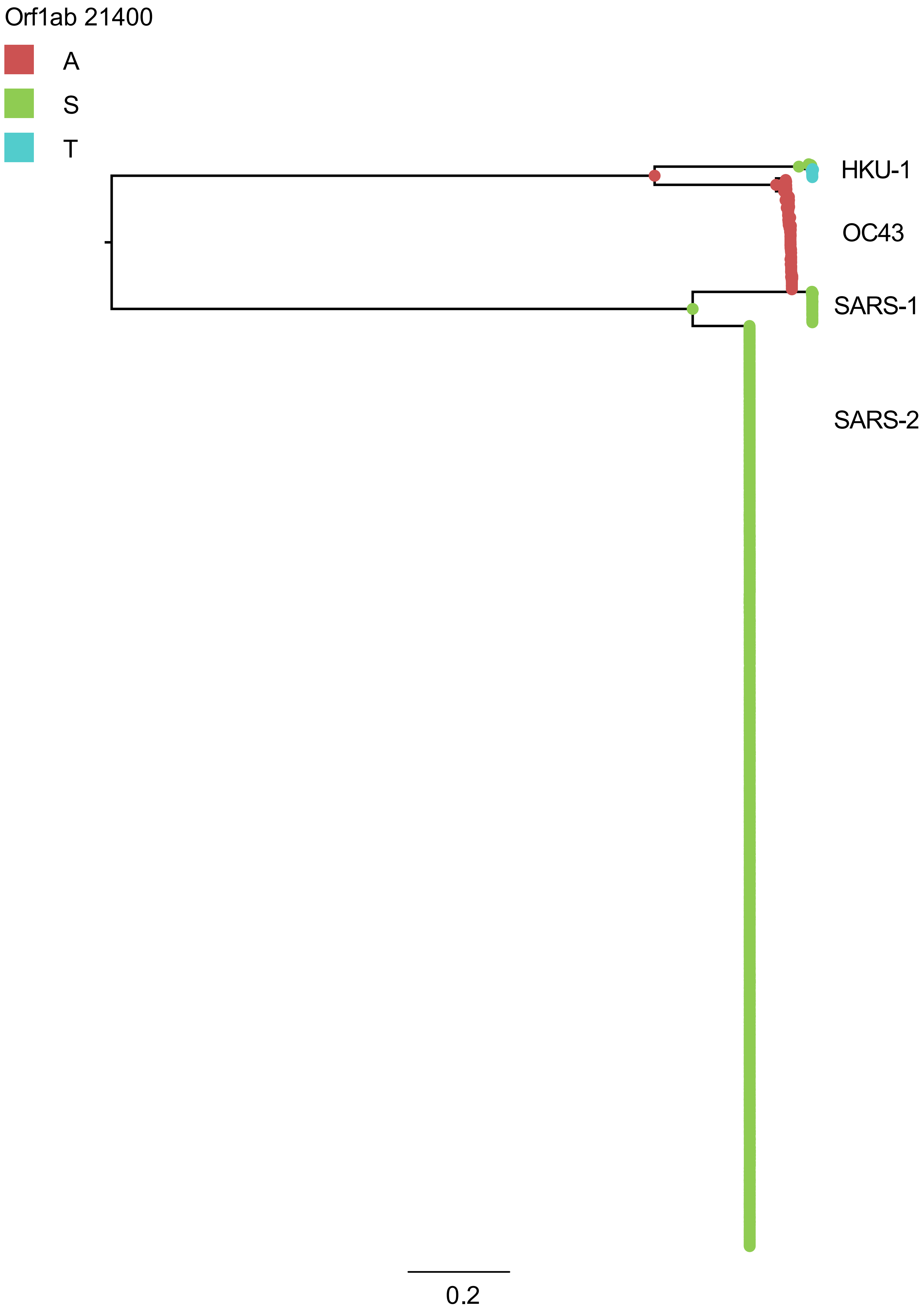
Orf1ab 21400
A
S
T
HKU-1
OC43
SARS-1
SARS-2
0.2

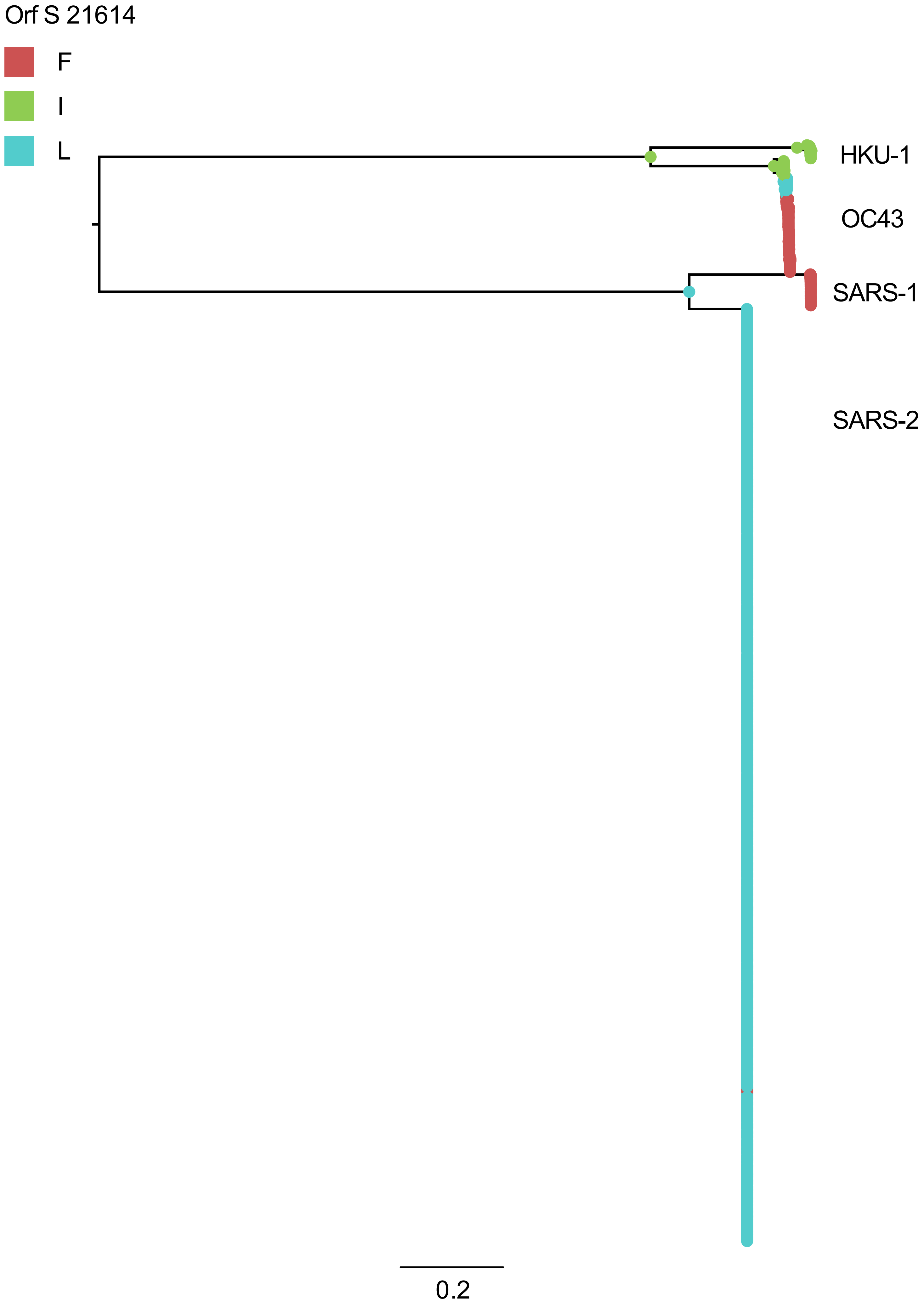
Orf S 21614
F
I
L
HKU-1
OC43
SARS-1
SARS-2
0.2

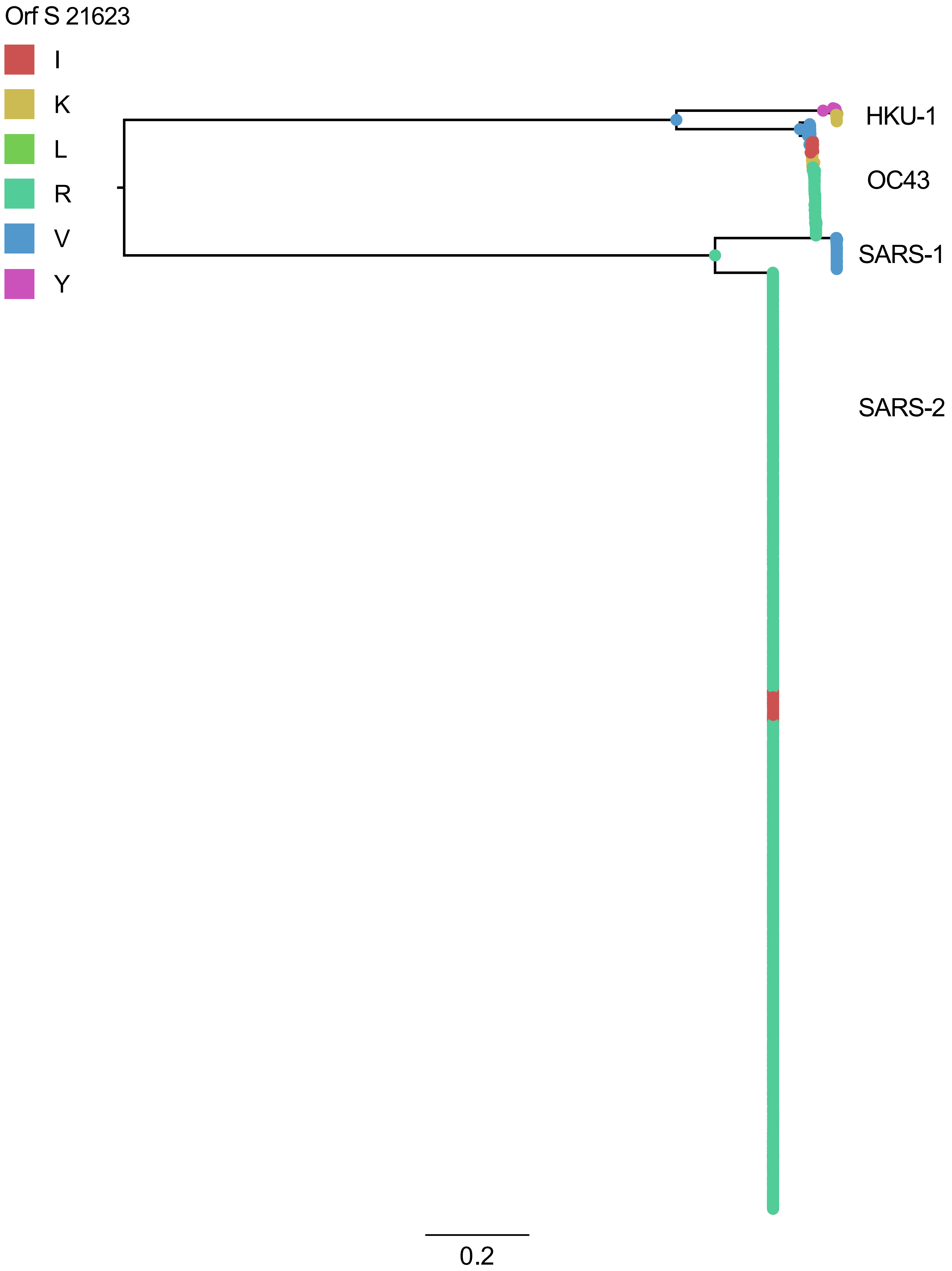

Orf S 21623
I
K
L
R
V
Y
HKU-1
OC43
SARS-1
SARS-2
0.2

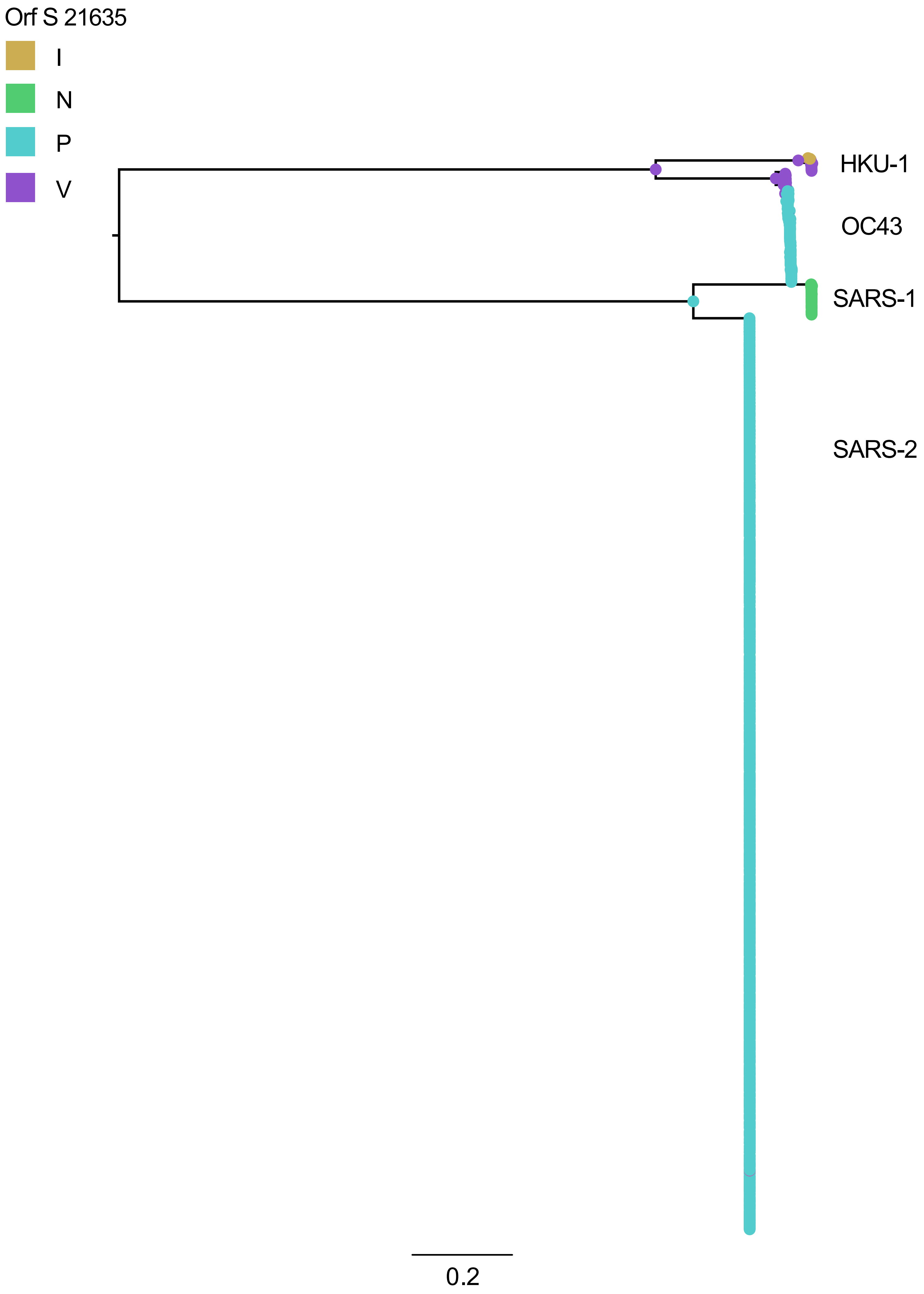

Orf S 21635
I
N
P
V
HKU-1
OC43
SARS-1
SARS-2
0.2

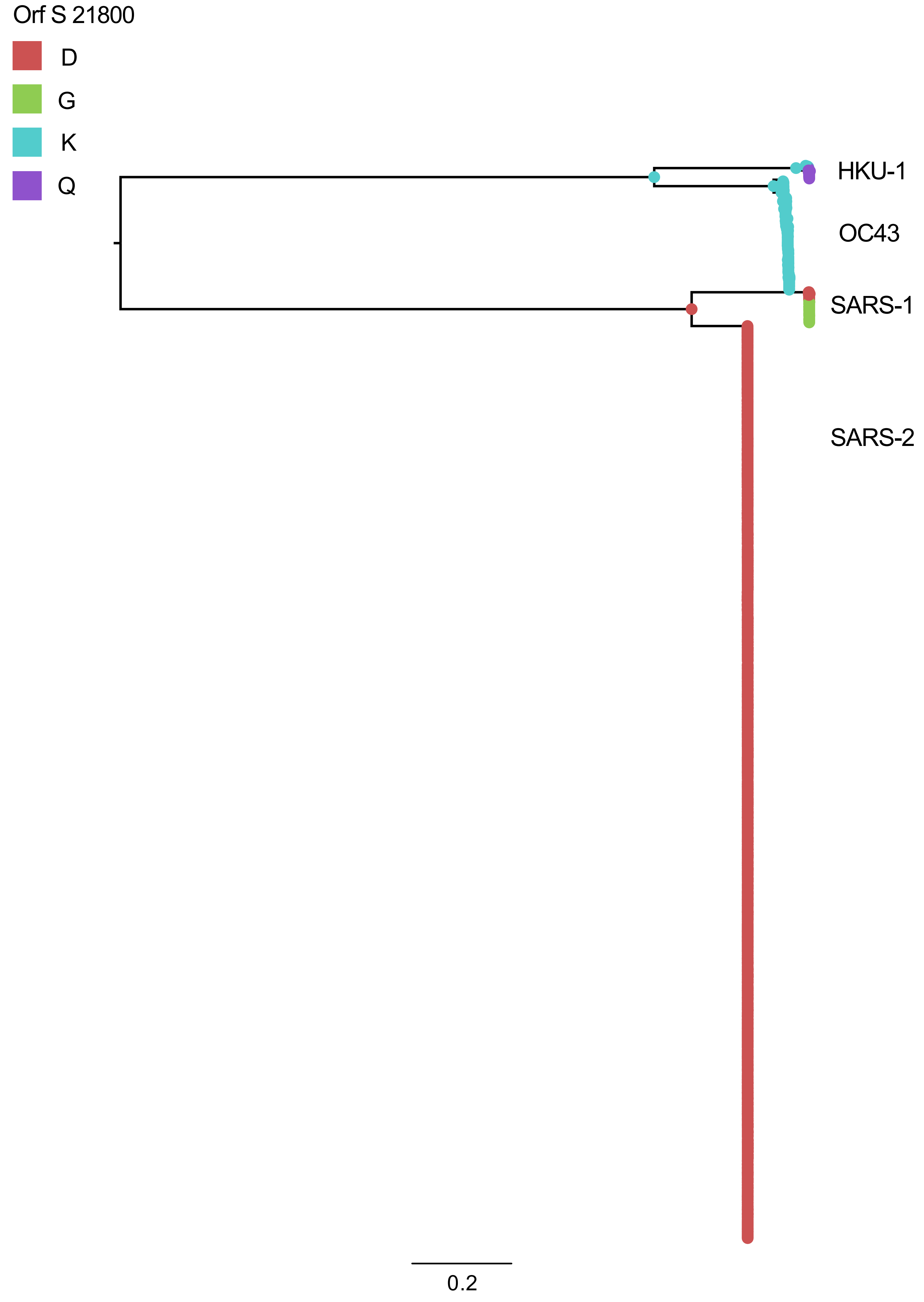
Orf S 21800
D
G
K
Q
HKU-1
OC43
SARS-1
SARS-2
0.2

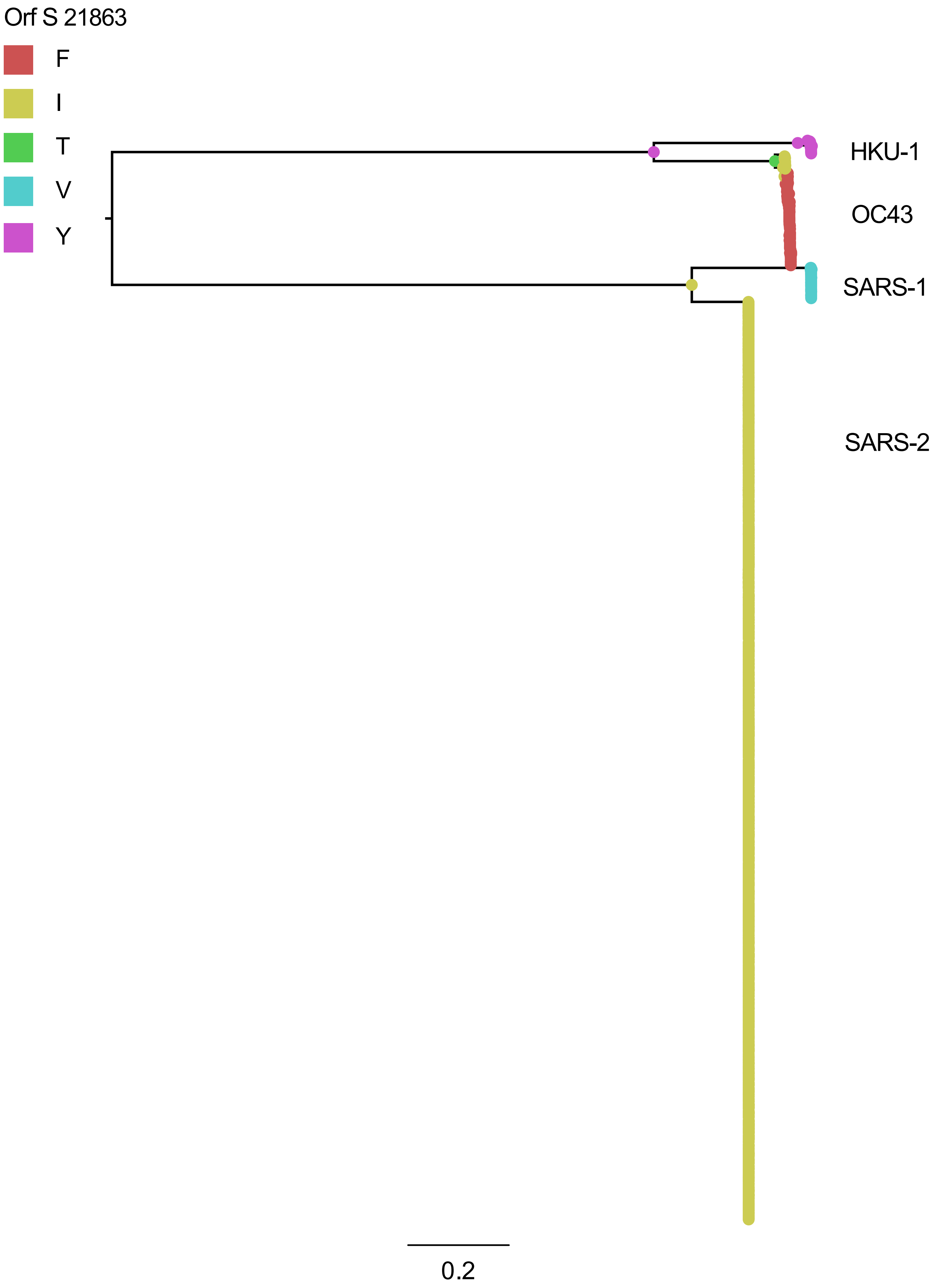

Orf S 21863
F
I
T
V
Y
HKU-1
OC43
SARS-1
SARS-2
0.2

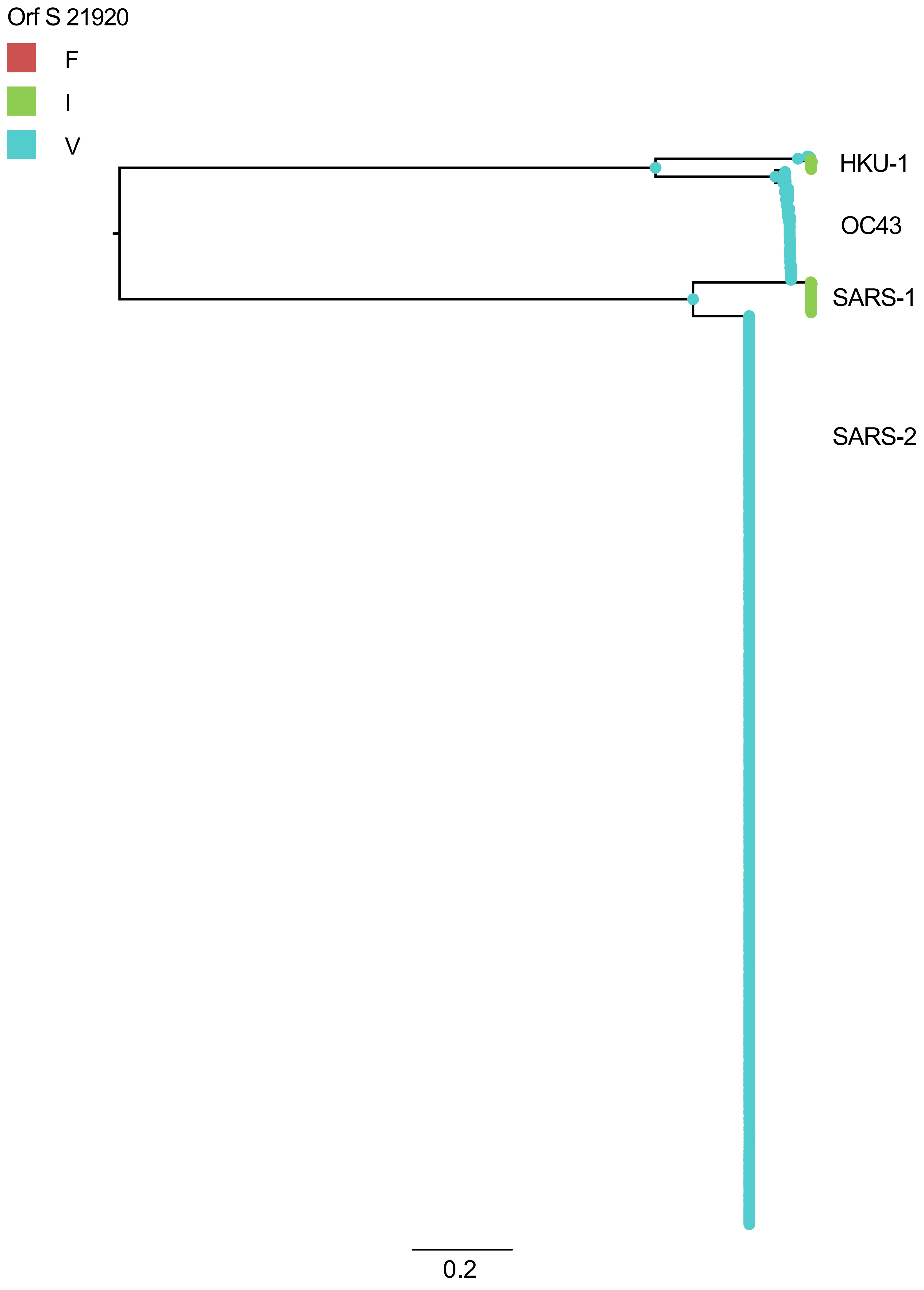

Orf S 21920
F
I
V
HKU-1
OC43
SARS-1
SARS-2
0.2

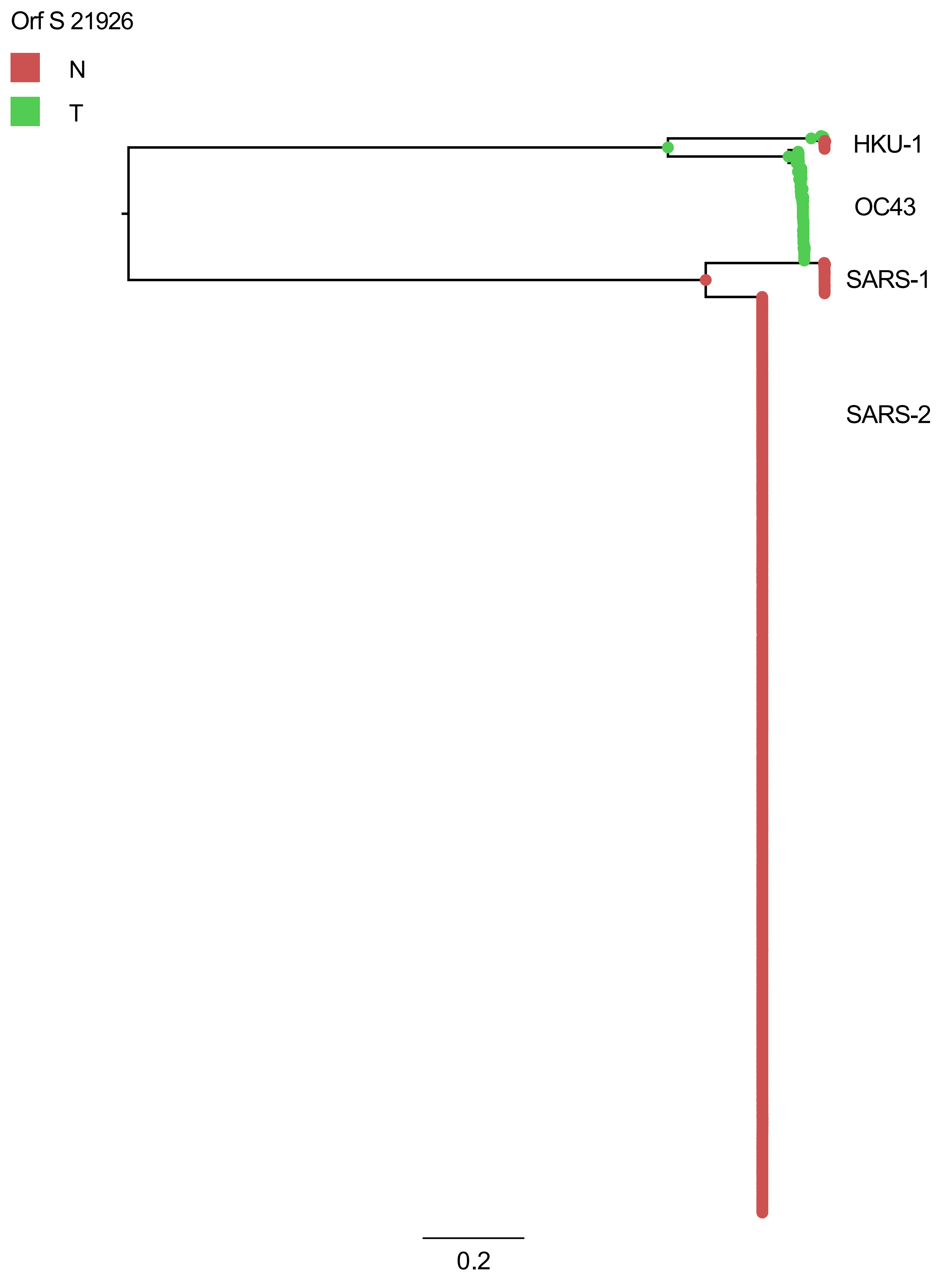

Orf S 21926
N
T
HKU-1
OC43
SARS-1
SARS-2
0.2

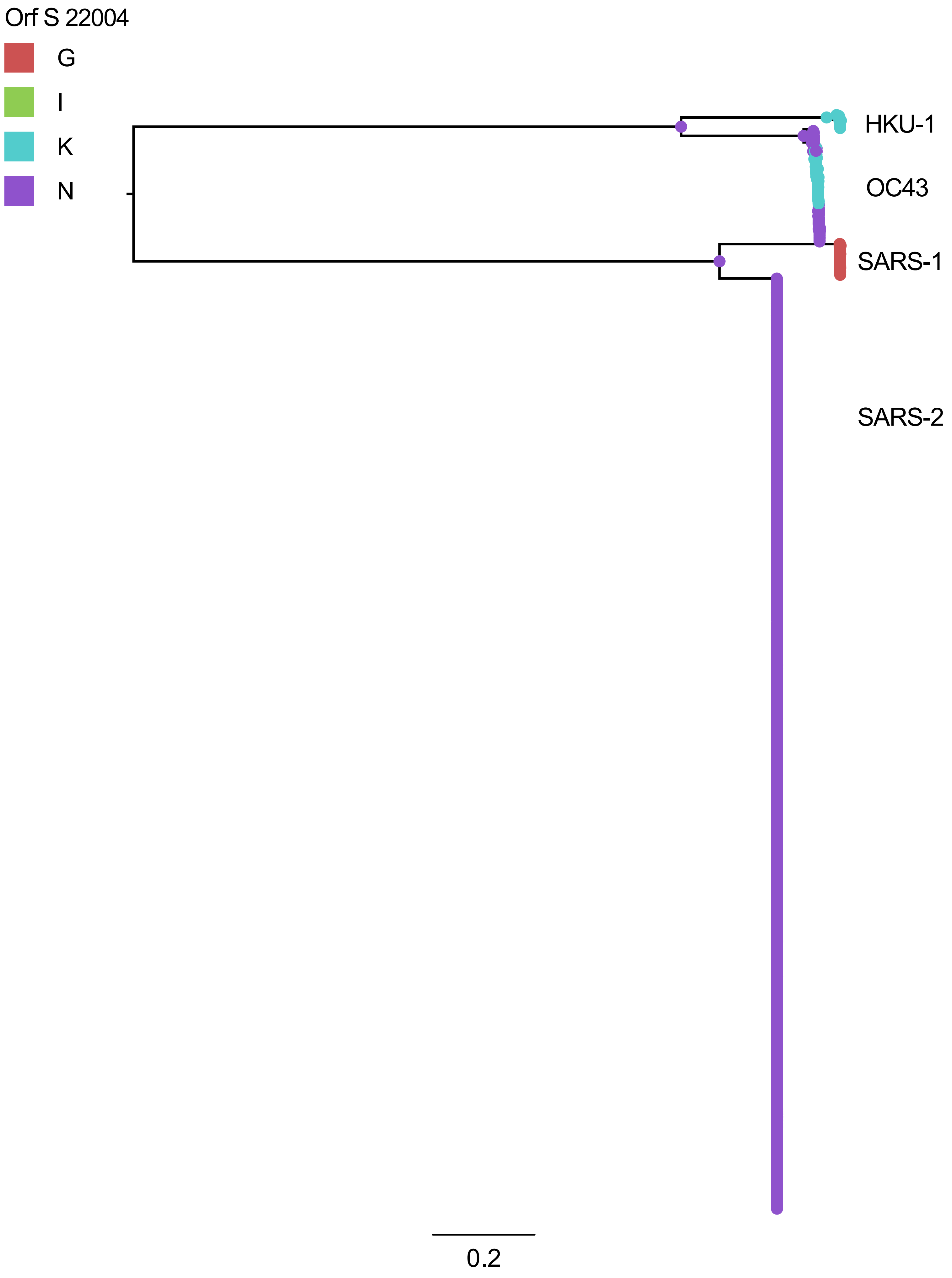
Orf S 22004
G
I
K
N
HKU-1
OC43
SARS-1
SARS-2
0.2

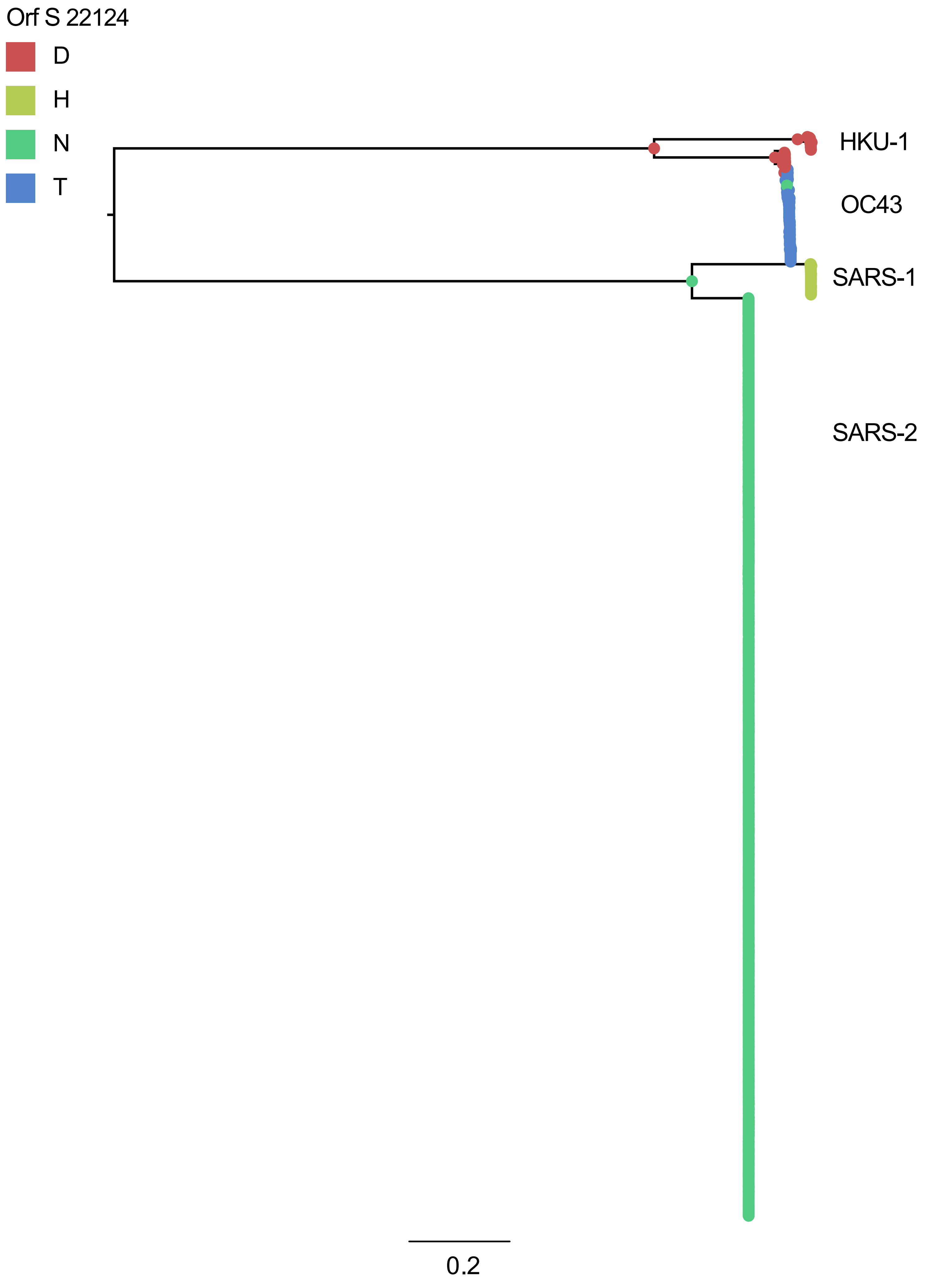
Orf S 22124
D
H
N
T
HKU-1
OC43
SARS-1
SARS-2
0.2

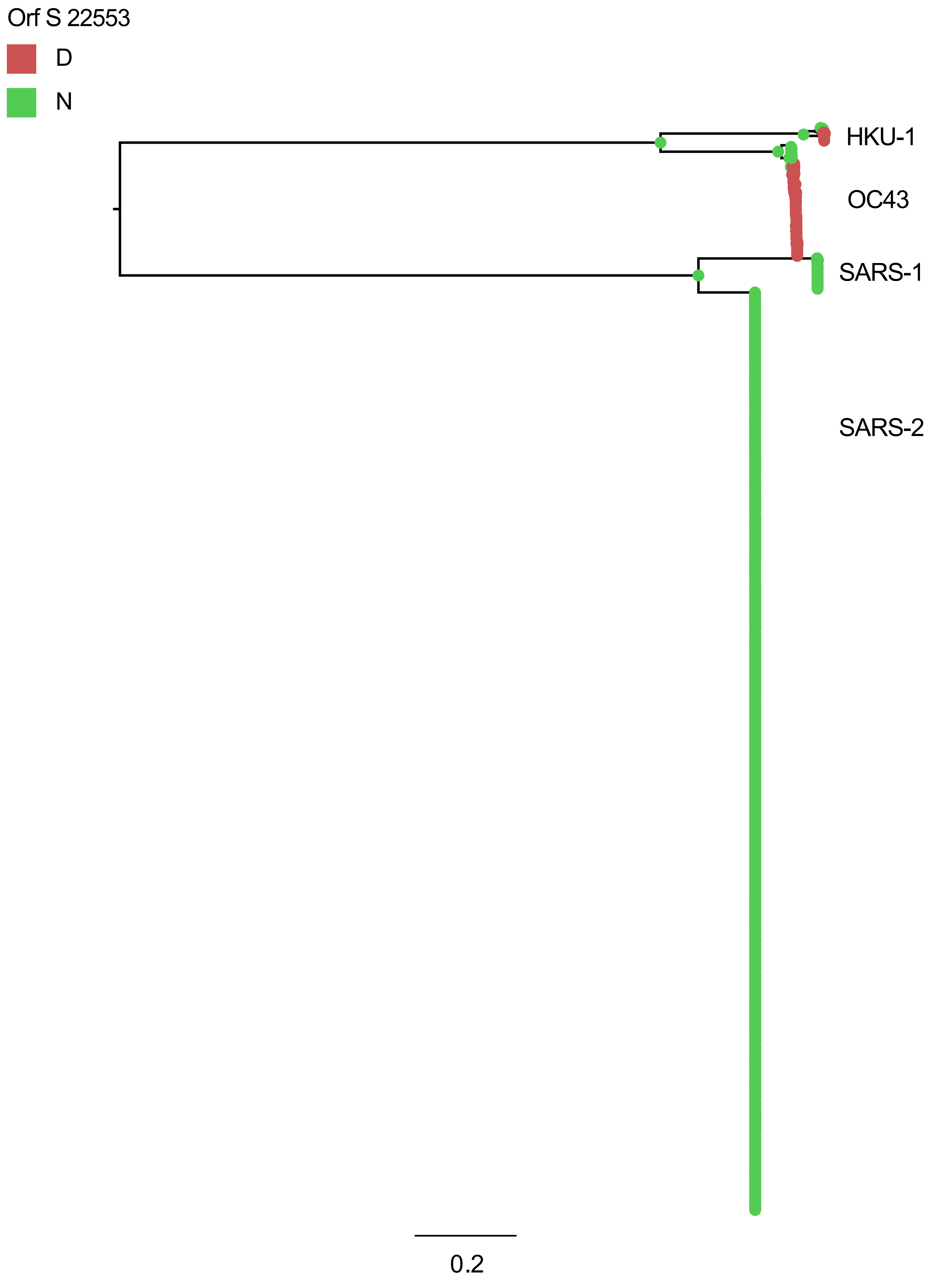
Orf S 22553
D
N
HKU-1
OC43
SARS-1
SARS-2
0.2

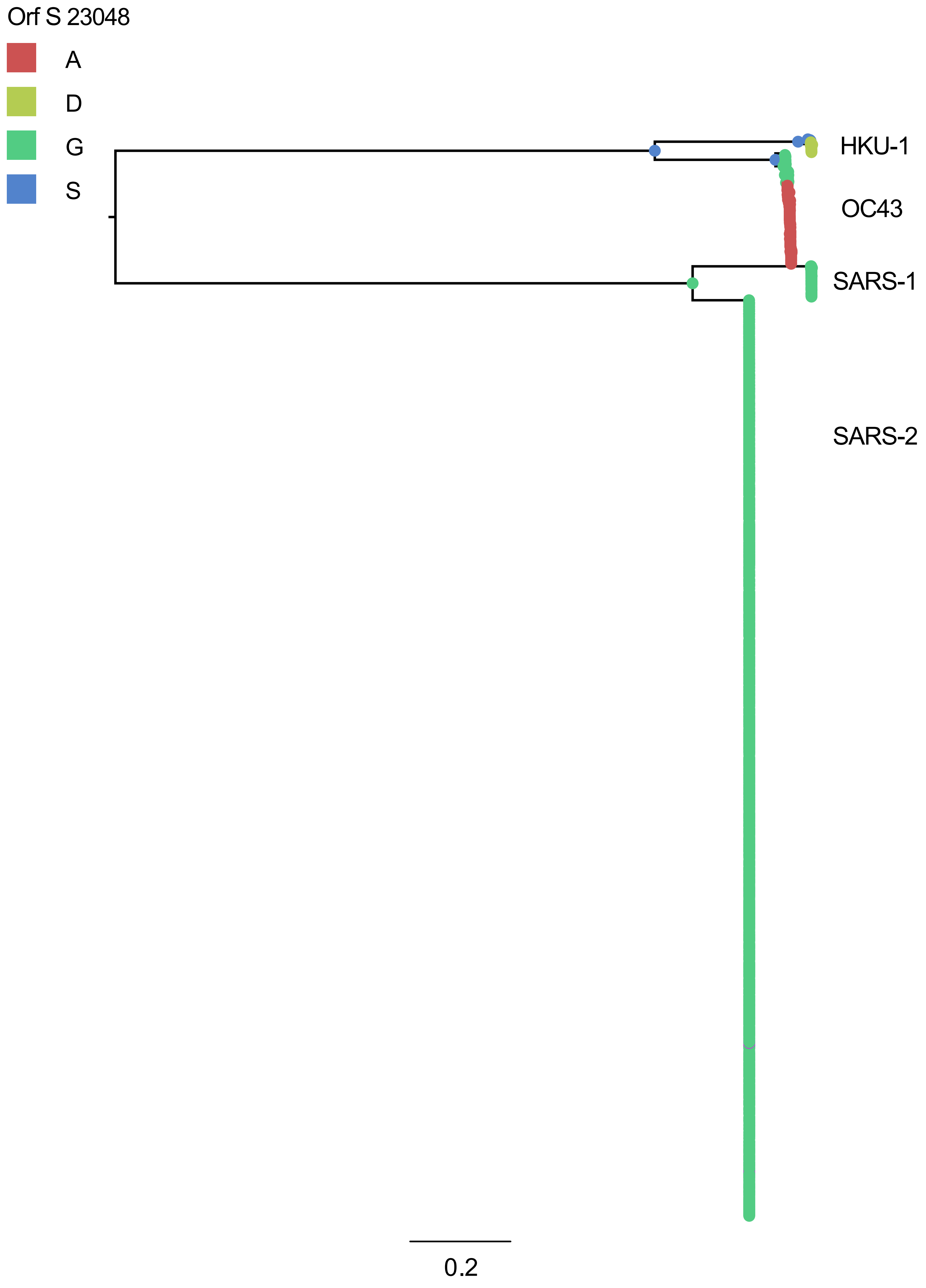

Orf S 23048
A
D
G
S
HKU-1
OC43
SARS-1
SARS-2
0.2

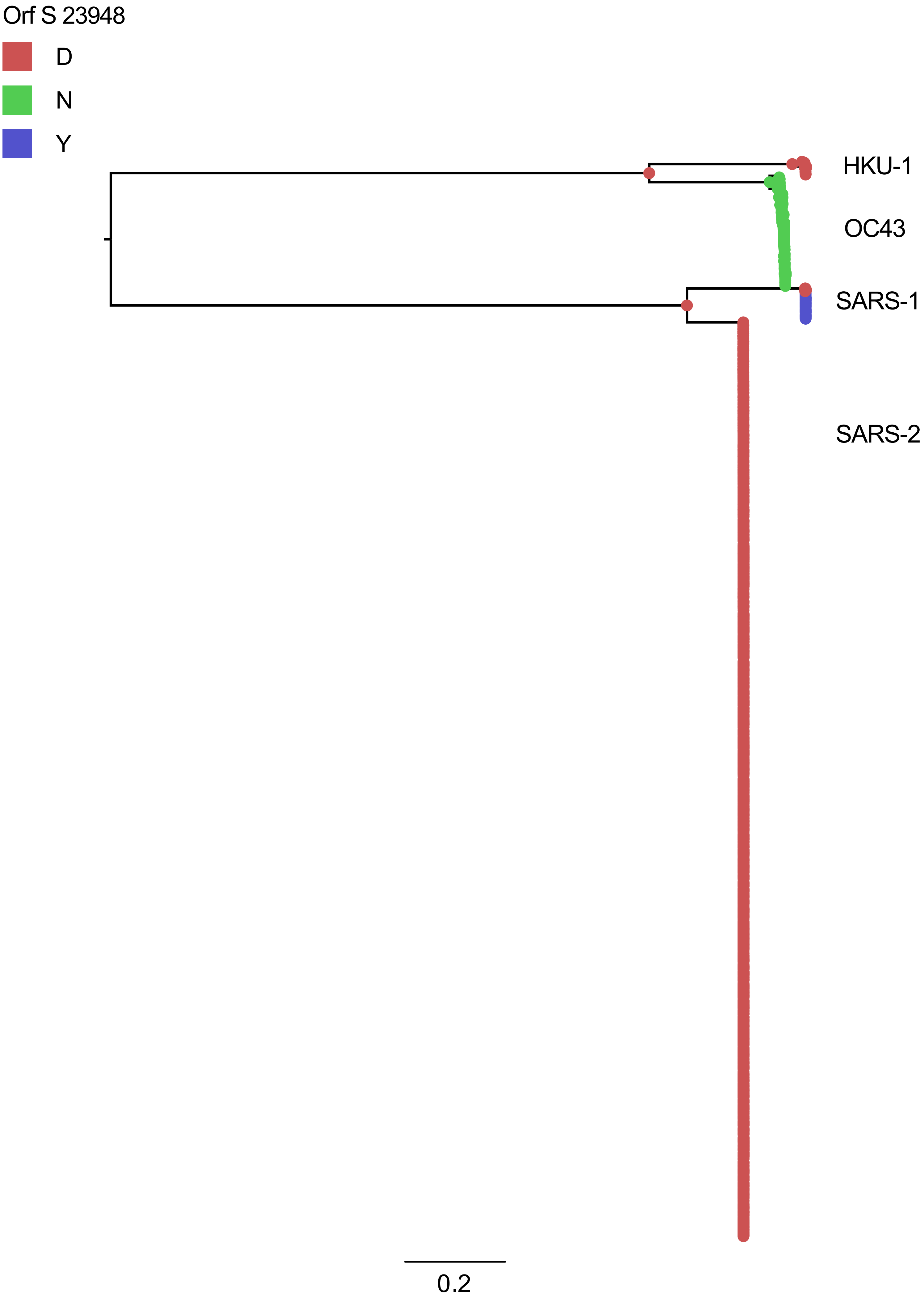

Orf S 23948
D
N
Y
HKU-1
OC43
SARS-1
SARS-2
0.2

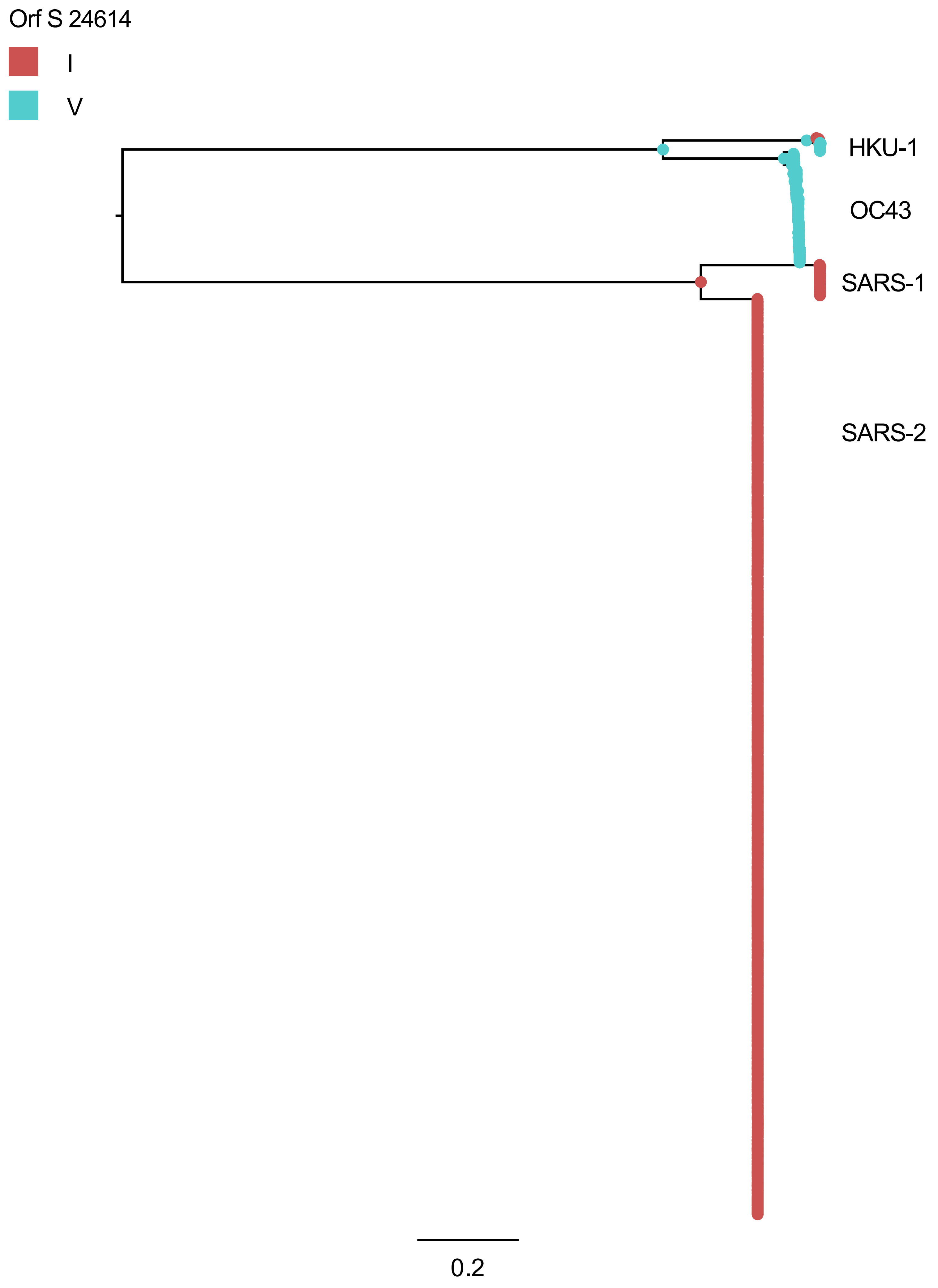
Orf S 24614
I
V
HKU-1
OC43
SARS-1
SARS-2
0.2

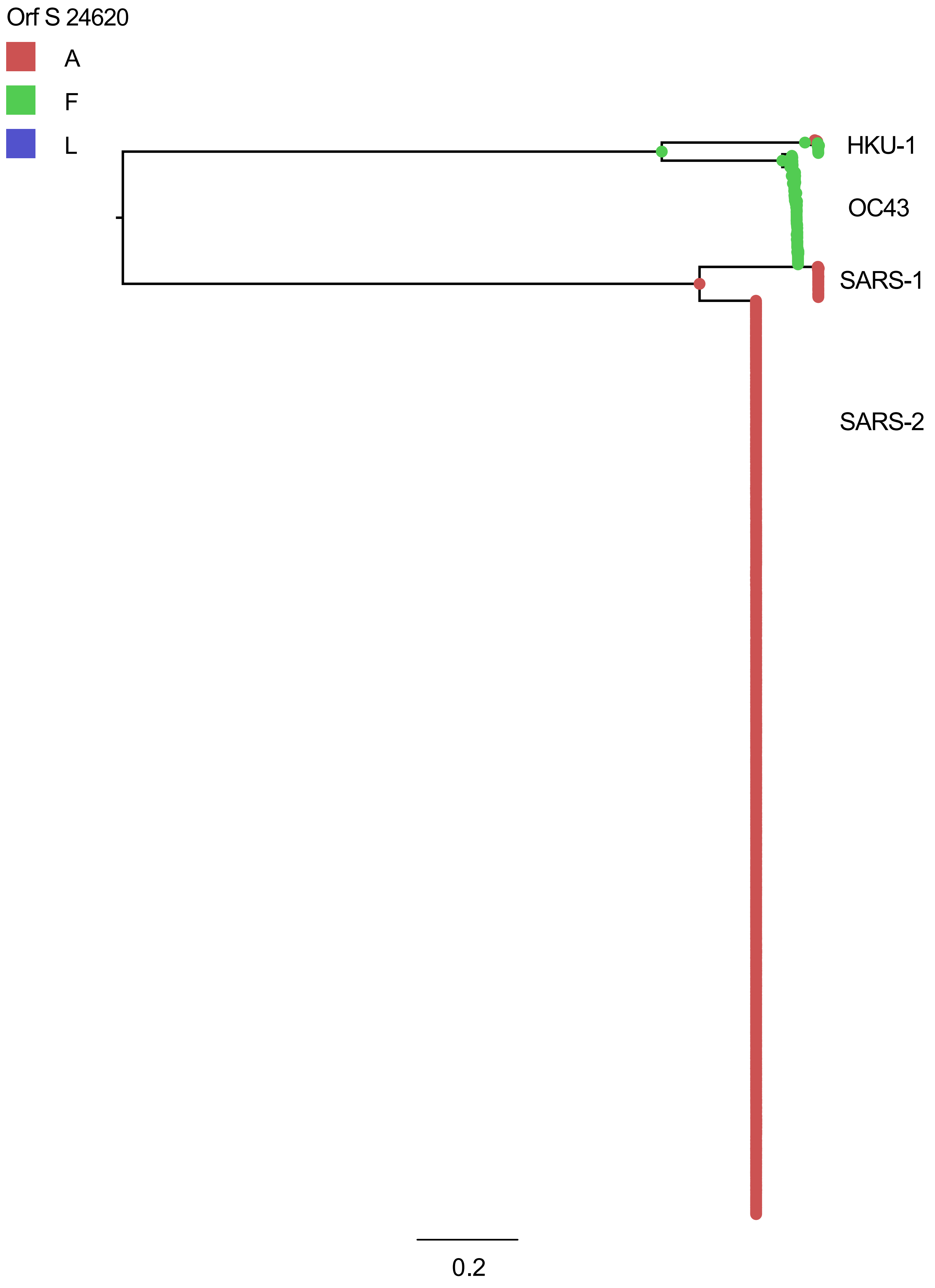
Orf S 24620
A
F
L
HKU-1
OC43
SARS-1
SARS-2
0.2

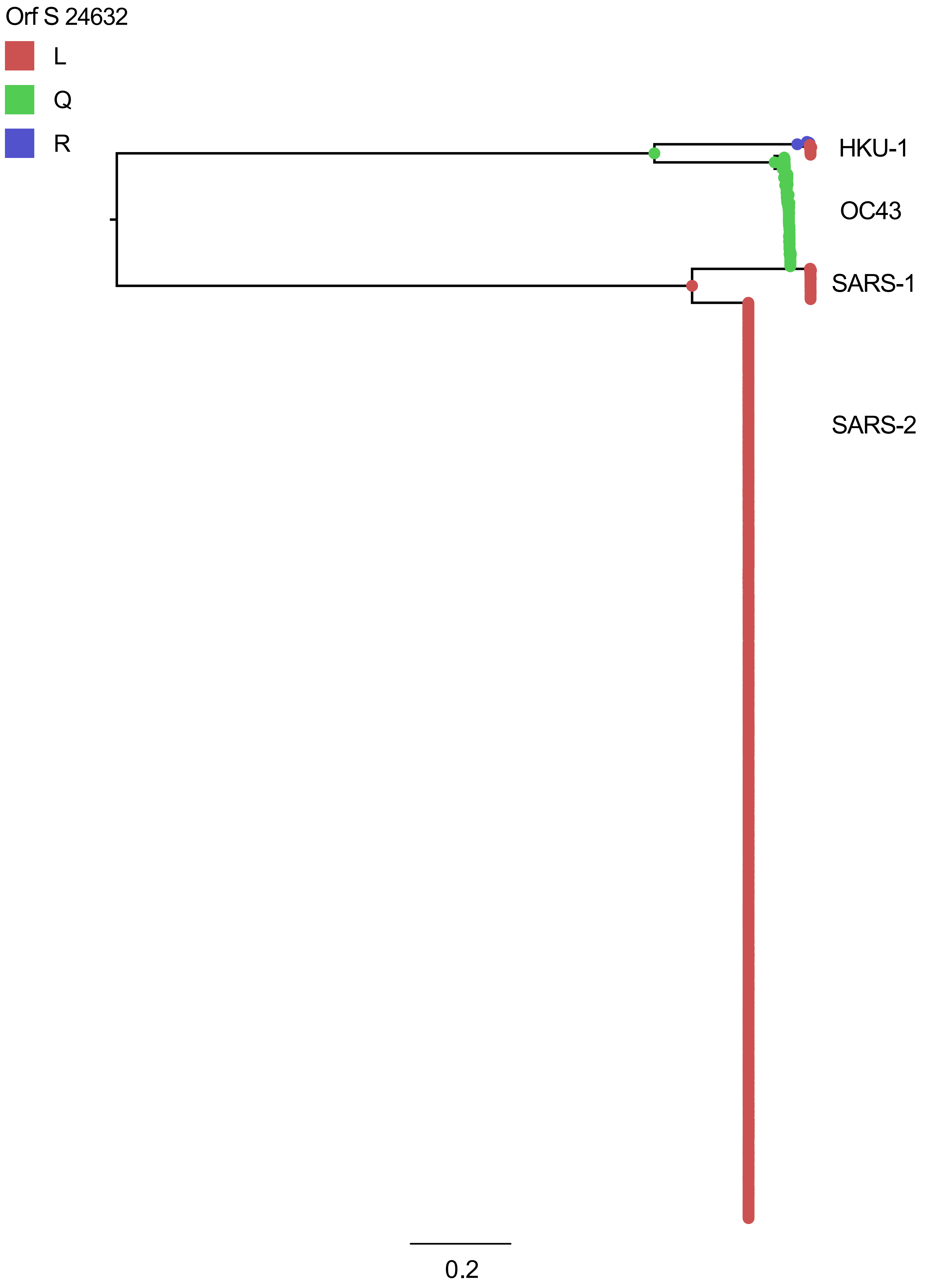
Orf S 24632
L
Q
R
HKU-1
OC43
SARS-1
SARS-2
0.2

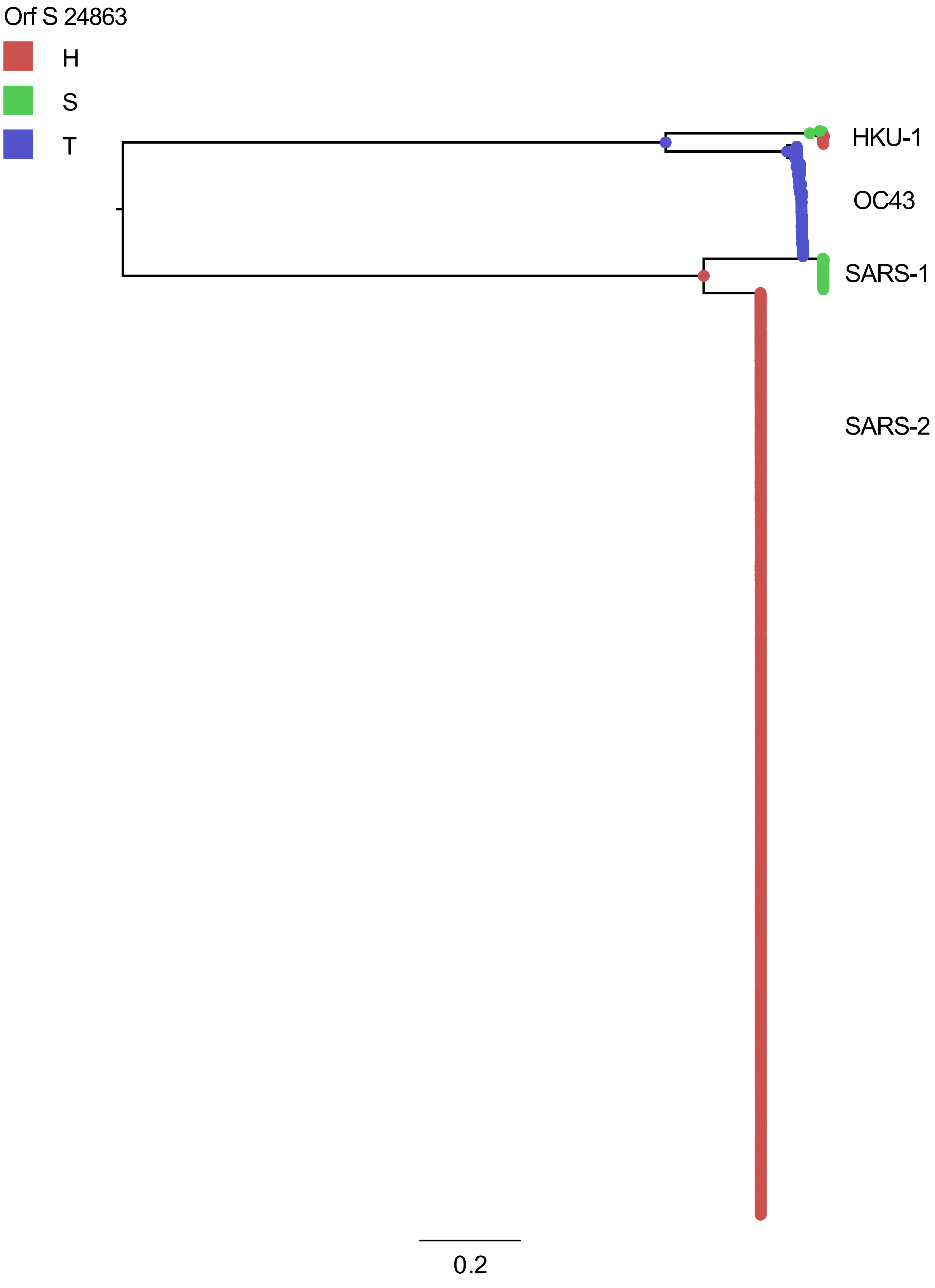
Orf S 24863
H
S
T
HKU-1
OC43
SARS-1
SARS-2
0.2

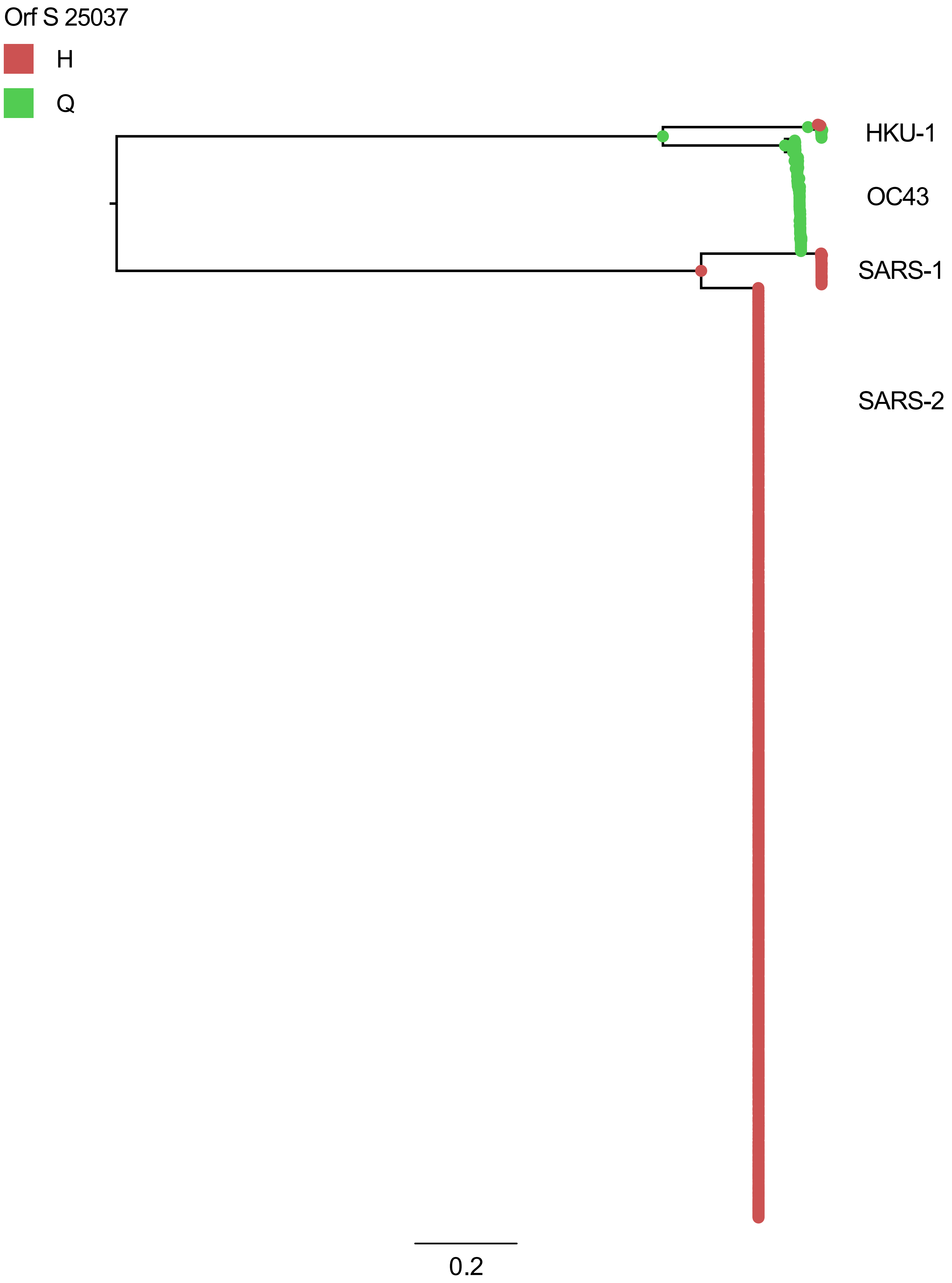
Orf S 25037
H
Q
HKU-1
OC43
SARS-1
SARS-2
0.2

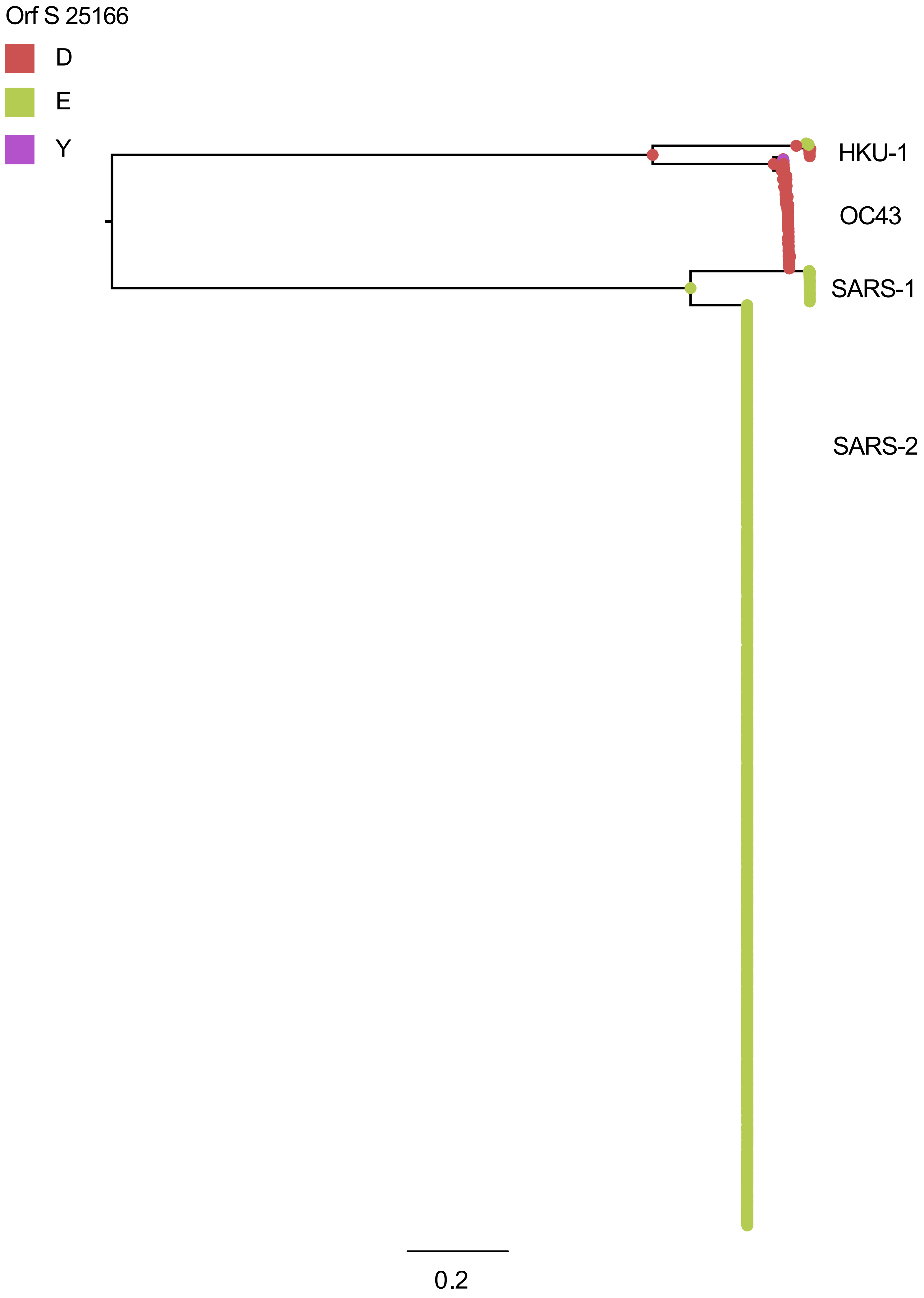

Orf S 25166
D
E
Y
HKU-1
OC43
SARS-1
SARS-2
0.2

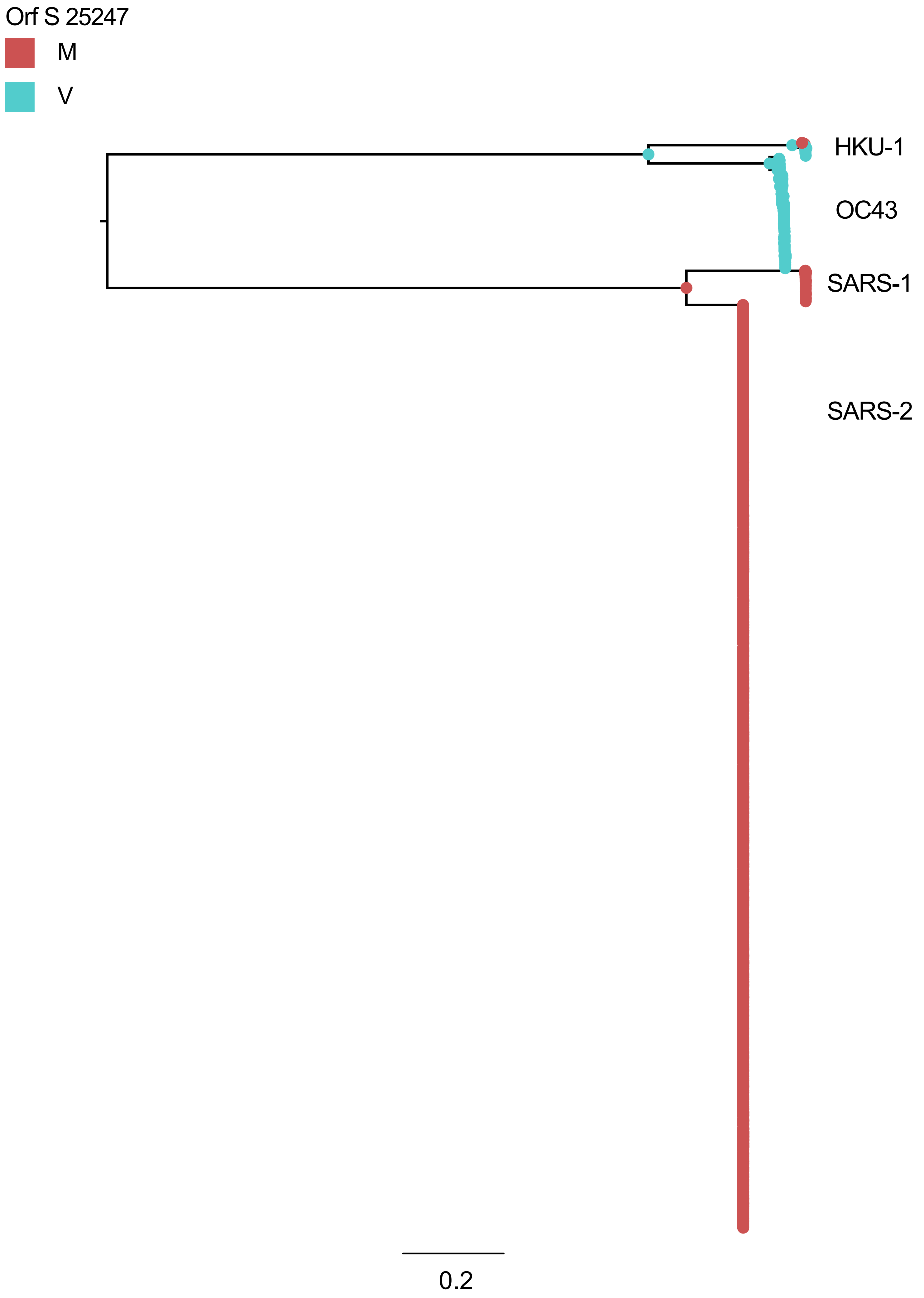
Orf S 25247
M
V
HKU-1
OC43
SARS-1
SARS-2
0.2
